# Teosinte-derived SynCom and precision biofertilization modulate the maize microbiome, enhancing growth, yield, and soil functionality in a Mexican field

**DOI:** 10.3389/fmicb.2025.1534327

**Published:** 2025-04-09

**Authors:** Juan Alfredo Hernández-García, Julio S. Bernal, Sanjay Antony-Babu, Lourdes Villa-Tanaca, César Hernández-Rodríguez, Esaú De-la-Vega-Camarillo

**Affiliations:** ^1^Laboratorio de Biología Molecular de Bacterias y Levaduras, Departamento de Microbiología, Escuela Nacional de Ciencias Biológicas, Instituto Politécnico Nacional, Ciudad de México, México; ^2^Department of Entomology, Texas A&M University, College Station, TX, United States; ^3^Department of Plant Pathology and Microbiology, Texas A&M University, College Station, TX, United States

**Keywords:** biofertilizer, SynCom, soil microbiome, precision agriculture, maize, teosinte, fungal diversity, bacterial diversity

## Abstract

Modern agriculture faces the challenge of optimizing fertilization practices while maintaining soil resilience and microbial diversity, both critical for sustainable crop production. We evaluated the effects of multiple fertilization strategies on soil microbial communities and plant performance, comparing conventional methods (urea-based and phosphorus fertilizers applied manually or via drone-assisted precision delivery) with biofertilization using a synthetic microbial consortium (SynCom) derived from teosinte-associated microbes. This SynCom consisted of seven bacterial strains: *Serratia nematodiphila* EDR2, *Klebsiella variicola* EChLG19, *Bacillus thuringiensis* EML22, *Pantoea agglomerans* EMH25, *Bacillus thuringiensis* EBG39, *Serratia marcescens* EPLG52, and *Bacillus tropicus* EPP72. High-throughput sequencing revealed significant shifts in bacterial and fungal communities across treatments. Untreated soils showed limited diversity, dominated by *Enterobacteriaceae* (>70%). Conventional fertilization gradually reduced *Enterobacteriaceae* while increasing *Pseudomonas* and *Lysinibacillus* populations. Drone-assisted conventional fertilization notably enhanced *Acinetobacter* and *Rhizobiales* growth. Biofertilization treatments produced the most pronounced shifts, reducing Enterobacteriaceae below 50% while significantly increasing beneficial taxa like *Bacillus, Pantoea*, and *Serratia*. Network analysis demonstrated that microbial interaction complexity increased across treatments, with *Bacillus* emerging as a keystone species. Drone-assisted biofertilization fostered particularly intricate microbial networks, enhancing synergistic relationships involved in nutrient cycling and biocontrol, though maintaining the stability of these complex interactions requires careful monitoring. Our findings provide key insights into how precision biofertilization with teosinte-derived microbial consortia can sustainably reshape the maize microbiome, improving crop performance and soil resilience.

## 1 Introduction

Integrating technological advancements empowered by automation into agricultural practices has garnered significant attention in recent years due to their potential to enhance production efficiency and sustainability (Pisante et al., 2012; Bellon Maurel and Huyghe, 2017; Pérez-Pons et al., 2020). Among these innovations, using drones to apply fertilizers and biofertilizers represents a promising advancement (Spoorthi et al., 2017). Traditional fertilizer application methods, characterized by uneven distribution and significant wastage, often result in suboptimal plant growth and exacerbate environmental issues such as nutrient leaching and soil degradation. Conversely, drone technology offers precision agriculture capabilities, ensuring uniform distribution and targeted application, which can minimize these drawbacks and improve overall agricultural productivity (Späti et al., 2021; Panjaitan et al., 2022; Maraveas, 2022). However, very little is known whether such automation practices have long-term effects on soil health that might influence agricultural productivity.

The soil microbiome is a significant soil health indicator, as it comprises a complex community of microorganisms, including bacteria, microbial eukaryotes, and archaea, and plays a crucial role in soil health and plant growth. These microorganisms are involved in essential processes such as nutrient cycling, organic matter decomposition, and disease suppression (Chaparro et al., 2012). However, conventional agricultural practices, including intensive chemical fertilizers, can disrupt these soil microbial communities, reducing soil fertility and increasing plant vulnerability to pests and diseases. Recent research has highlighted the importance of preserving and enhancing soil microbial diversity as a strategy for sustainable agriculture (Hartmann et al., 2015; Lupatini et al., 2017; Gupta et al., 2022).

Biofertilizers are formulations of living microorganisms that promote plant growth by increasing the availability of primary nutrients to the host plant and have emerged as an eco-friendly alternative to chemical fertilizers (Wu et al., 2005; Li et al., 2023). They offer numerous benefits, including improved soil structure, enhanced nutrient uptake, and increased resilience to environmental stressors (Bhardwaj et al., 2014). Biofertilizers applied through drone technology could amplify these benefits by ensuring precise and efficient delivery, thereby maximizing their positive impact on the soil microbiome (Malusá et al., 2012; Schütz et al., 2018; Mitter et al., 2021).

Despite several theoretical advantages, very few empirical studies have rigorously quantified the impact of drone-delivered fertilizers and biofertilizers on soil microbial community structure and function, particularly in field settings. Most research has mainly focused on the effects of these substances when applied through traditional methods (Wu et al., 2005; Nosheen et al., 2021). Any potential synergistic effects of combining drone technology with biofertilization practices on soil microbiota remain largely unexplored. Understanding these interactions is critical for developing innovative and sustainable soil management practices that can support the growing global demand for food while preserving environmental health (Malusá et al., 2012; Bamdad et al., 2022).

Recent studies have highlighted the potential of microbial consortia, particularly those derived from native plant species, in enhancing soil nutrient dynamics and plant resilience under various environmental conditions (Vassilev et al., 2015; Olanrewaju et al., 2017). In maize cultivation, biofertilizers containing *Bacillus, Pseudomonas*, and *Azospirillum* have been shown to improve root architecture, nitrogen fixation, and phosphate solubilization, leading to higher yields and improved soil structure (Bhattacharyya and Jha, 2012; Singh et al., 2020). However, challenges remain in ensuring the persistence and colonization of these beneficial microbes in agricultural soils, particularly when applied through mechanized or precision-based approaches (Compant et al., 2010). Recent advances suggest that the integration of biofertilization with emerging technologies, such as drone-assisted delivery, could optimize microbial survival and function by ensuring even distribution and minimizing environmental stressors during application (de Souza et al., 2021). Despite these theoretical advantages, field-based evidence remains limited, necessitating further research into how precision application techniques influence microbial community dynamics and long-term soil health (Bashan et al., 2014; Hartmann et al., 2015).

This study aimed to fill this knowledge gap by investigating the interacting effects of traditional and drone-based chemical fertilizer and biofertilizer application methods on the soil microbiome. Through high-throughput sequencing and comprehensive microbial analysis, we examined how these application strategies influence soil microbial community composition, diversity, and functionality. The findings will provide valuable insights into the potential of drone technology to enhance soil health and promote sustainable agricultural practices.

## 2 Materials and methods

### 2.1 Field site selection and experimental design

This study was conducted in native Mexican maize fields in the rural community of San Juan de las Manzanas, Ixtlahuaca de Rayón, Estado de México (−99.842025 and 19.556658). The experimental plots were established using a randomized complete block design (RCBD) with five replicates per treatment (Piepho et al., 2004). Each plot received one of the following treatments: no application (control) (NA), conventional fertilization backpack (MF) and drone-assisted (DF), and biofertilization with SynCom applied manually (MB) and via drone technology (DB) (Mogili and Deepak, 2018; Saleem et al., 2019). Comprehensive site characterization was performed for each field to account for environmental variability. This included soil type classification (IUSS Working Group WRB, 2015), collection of historical crop data, and recording climatic conditions throughout the growing season (Lobell et al., 2011). These environmental parameters were integrated into subsequent data analyses to assess treatment efficacy across diverse agroecological contexts.

### 2.2 Biofertilizer (SynCom) formulation

Based on functional traits, SynCom enhanced plant growth, nutrient cycling, and pathogen suppression. The formulation process involved screening bacterial isolates for nitrogen fixation, phosphate solubilization, and production of phytohormones such as indole-3-acetic acid (IAA). The SynCom comprised seven beneficial bacterial strains isolated from teosinte seeds: *Serratia nematodiphila* EDR2, *Klebsiella variicola* EChLG19, *Bacillus thuringiensis* EML22, *Pantoea agglomerans* EMH25, *Bacillus thuringiensis* EBG39, *Serratia marcescens* EPLG52, and *Bacillus tropicus* EPP72 (Table 1) (De-la-Vega-Camarillo et al., 2023a). Each bacterial strain was cultured independently in R2A broth media at 28°C with orbital shaking at 150 rpm until reaching an optical density (OD600) of 1.0 (Reasoner and Geldreich, 1985). Cultures were then centrifuged at 13,000 rpm for 10 min, and the resulting pellets were resuspended in sterile saline solution (0.85% NaCl) to achieve a final concentration of 1 × 10^9^ CFU/ML (De-la-Vega-Camarillo et al., 2023b). The SynCom was prepared for field application by mixing equal volumes of each bacterial suspension. The viability of each strain in the final mixture was confirmed through colony-forming unit (CFU) counts on selective media (Timmusk et al., 2017).

**Table 1:** Bacterial strains isolated from *Zea* species and their functional traits.

**Strain**	**Source**	**Relevant phenotype**
*Serratia nematodiphila* EDR2	*Zea diploperennis*	Amylase
*Klebsiella variicola* EChLG19	*Zea mays* subsp. mexicana Chalco landrace	Metalophores 6 ions (Fe, Mo, Cu, V, Co, and Zn)
*Bacillus thuringiensis* EML22	*Zea mays* subsp. mexicana Mesa Central landrace	Phosphate solubilization, IAA
*Pantoea agglomerans* EMH25	*Zea mays* subsp. mexicana Mesa Central landrace	ACC deaminase
*Bacillus thuringiensis* EBG39	*Zea mays* subsp. Parviglumis Balsas landrace	BFN, Chitinase
*Serratia marcescens* EPLG52	*Zea perennis*	Protease
*Bacillus tropicus* EPP72	*Zea perennis*	Cellulase

### 2.3 Application of treatments

The treatments were applied using drone-assisted and backpack methods. For drone application, a DJI Agras MG-1S drone equipped with four precision nozzles was utilized (Zhang and Kovacs, 2012). The drone was calibrated to deliver 50 mL of treatment solution per plant, with flight paths programmed to ensure uniform coverage of each plot (Tripicchio et al., 2015). Backpack applications were conducted using backpack sprayers, calibrated to deliver 50 mL per plant, following standard agronomic practices (Szilagyi-Zecchin et al., 2016). Applications were carried out at four key growth stages: pre-treatment at 0 days after sowing (DAS), mid-season at 25 DAS, early reproductive stage at 55 DAS, and harvest at 85 DAS, aligning with critical periods in maize development (Abendroth et al., 2011). Weather conditions, including temperature, humidity, and wind speed, were recorded during each application to account for potential variability in treatment efficacy (Sánchez et al., 2014). These environmental parameters were later incorporated into the data analysis to assess their impact on treatment performance.

### 2.4 Soil sampling

Soil samples were collected from each experimental plot 5 days after the application to observe the changes in the microbial communities due to fertilization and biofertilization processes as follows: 0 DAS (pre-treatment), 30 DAS (mid-season), 60 DAS (early reproductive stage), and 90 DAS (post-harvest) (Ma and Biswas, 2015). Each sample comprised five subsamples taken from the corners and center of each plot at a depth of 0–15 cm using a sterilized soil drill (Peigné et al., 2013). The subsamples were homogenized to create a composite sample (~500 g) per plot, following standard soil sampling protocols (Larkin, 2015). Immediately after collection, soil samples were placed in sterile, airtight containers and transported on ice to the laboratory. To preserve microbial community structure and DNA integrity, samples were stored at −80°C until further processing (Rissanen et al., 2010).

### 2.5 Physicochemical soil analysis

Soil physicochemical properties were analyzed following standardized protocols. Before analysis, samples were air-dried at room temperature (25 ± 2°C), ground, and sieved through a 2-mm mesh to remove coarse particles and plant debris (International Organization for Standardization, 2006). The following parameters were assessed: pH (1:2.5 soil/water ratio), electrical conductivity (EC), organic matter content (Walkley-Black method), total nitrogen (Kjeldahl method), available phosphorus (Olsen method), and exchangeable potassium (ammonium acetate extraction) (Bao, 2000; Jones, 2001).

### 2.6 Spectral data collection and analysis

Soil nutrient availability was assessed using a combination of satellite and drone-based multispectral imaging. Satellite data were obtained from Sentinel-2 MSI (10 m resolution) (Drusch et al., 2012). At the same time, high-resolution imagery (3 cm/pixel) was acquired using a DJI Phantom 4 Multispectral drone equipped with a six-band multispectral camera (blue, green, red, red edge, near-infrared, and RGB) (Hassan et al., 2019). Drone flights were conducted at 40 m altitude between 10:00 and 14:00 under clear sky conditions (cloud cover < 10%), coinciding with soil sampling dates (Assmann et al., 2019). Radiometric calibration was performed using a calibrated reflectance panel before each flight (Wang and Myint, 2015). Both satellite and drone imagery were atmospherically corrected using Sen2Cor (version 2.8) and Pix4D Mapper (version 4.6.4), respectively (Main-Knorn et al., 2017; Pix4D SA, 2020).

Spectral indices were calculated to estimate specific soil nutrients: Modified Soil-Adjusted Vegetation Index (MSAVI) for nitrogen content, Band Ratio Phosphorus Index (BRPI) for available phosphorus, and Potassium Abundance Index (KAI) for exchangeable potassium (Wang et al., 2014; Song et al., 2023). Additional indices included the Iron Oxide Ratio (IOR) and Normalized Difference Salinity Index (NDSI) for micronutrient availability assessment (Ge et al., 2011). Calibration models were developed using partial least squares regression (PLSR) to relate spectral indices to laboratory-measured nutrient concentrations (Viscarra Rossel and Behrens, 2010). Model validation was performed using a subset of soil samples (30%) that was not used in calibration.

For each experimental plot, spectral data were extracted from both satellite (10 m^2^ pixels) and drone imagery (9 cm^2^ pixels) using zonal statistics in QGIS 3.22 (QGIS Development Team, 2024). Temporal variations in nutrient availability were analyzed using repeated measures ANOVA with Bonferroni correction for multiple comparisons (*p* < 0.05). The relationship between spectral-derived and laboratory-measured nutrient values was assessed using Spearman correlation coefficients and root mean square error (RMSE) calculations (Gorelick et al., 2017).

### 2.7 DNA extraction and metagenomic analysis

Total genomic DNA was extracted from 0.5 g of soil using the DNeasy PowerSoil Kit (Qiagen, Hilden, Germany), following the manufacturer's instructions with an additional bead-beating step for enhanced cell lysis (Lim et al., 2010). The V3–V4 regions of the 16S rRNA gene were amplified using 341F/785R (CCTACGGGNGGCWGCAG/GACTACHVGGGTATCTAATCC) primers for bacteria (Klindworth et al., 2013), and the internal transcribed spacer (ITS) regions were amplified using ITS1F/ITS2 (CTTGGTCATTTAGAGGAAGTAA/GCTGCGTTCTTCATCGATGC) primers for fungi (Gardes and Bruns, 1993); both with unique 6-nucleotide barcodes for sample identification. PCR reactions were performed in 30 μL volumes containing 15 μL Phusion Master Mix (New England Biolabs, Ipswich, MA, USA), 0.2 μm of each primer, and 10 ng of template DNA. The thermal cycling conditions were as follows: initial denaturation at 98°C for 1 min, followed by 30 cycles of 95°C for 10 s, 50°C for 30 s (16S primers)/56°C for 30 s (ITS primers), and 72°C for 30 s, with a final extension at 72°C for 5 min (Wu et al., 2015). Amplified products were purified using the GeneJET Gel Extraction Kit (Thermo Fisher Scientific, Waltham, MA, USA) and verified by 2% agarose gel electrophoresis. Sequencing was performed on the Illumina MiSeq platform at Genome Science Core, Wayne State University (Detroit, USA), with a read length of 250 bp paired-end (Caporaso et al., 2011).

### 2.8 Bioinformatic processing

Raw sequencing data were processed using QIIME2 (version 2021.2) for demultiplexing and quality filtering (Bolyen et al., 2019). Reads with a Phred score below 20, chimeric sequences, and those shorter than 200 bp were discarded. Operational taxonomic units (OTUs) were delineated at 97% sequence similarity using DADA2 (Callahan et al., 2016). Taxonomic identities were attributed in comparison to the SILVA database (v13.8) for bacterial sequences (Quast et al., 2012) and the UNITE database (v132) for fungal sequences (Nilsson et al., 2019).

Microbial community diversity was assessed using both alpha and beta diversity metrics. Alpha diversity was quantified using Shannon, Simpson, Chao1, ACE (Abundance-based Coverage Estimator) indices, and observed species richness, calculated using the scikit-bio library (scikit-bio Development Team, 2020) in Python 3.8 (Van Rossum and Drake, 2009). Rarefaction curves were generated to evaluate sampling depth adequacy using the rarefaction_curve function from scikit-bio. Beta diversity was assessed using weighted and unweighted UniFrac distances (Lozupone et al., 2011), Bray-Curtis dissimilarity index (Bray and Curtis, 1957), Jaccard index (Jaccard, 1912), and Hellinger distance (Legendre and Gallagher, 2001), computed with the scipy.spatial.distance module (Virtanen et al., 2020). To visualize relationships between microbial communities, we performed Principal Coordinate Analysis (PCoA) and Non-metric Multidimensional Scaling (NMDS) using the scikit-learn library (Pedregosa et al., 2011). Differences in alpha diversity metrics between treatment groups were assessed using one-way ANOVA followed by Tukey's HSD *post-hoc* test, implemented with the scipy—stats module. For beta diversity, the statistical significance of community differences was tested using Permutational Multivariate Analysis of Variance (PERMANOVA) with 999 permutations, implemented through the skbio.stats.distance module. To elucidate potential microbial interactions, co-occurrence networks were constructed using three complementary approaches: the SparCC algorithm (Friedman and Alm, 2012) implemented in the FastSpar package (Watts et al., 2019), the CoNet method (Faust and Raes, 2016) using the RMT-based approach in the MENA package (Deng et al., 2012), and SPIEC-EASI (Kurtz et al., 2015) using the SpiecEasi Python package. Networks were visualized using NetworkX (Hagberg et al., 2008) and plotted with Matplotlib (Hunter, 2007). Network properties, including modularity, average path length, and clustering coefficient, were calculated using NetworkX. To identify microbial taxa significantly enriched or depleted between different treatments, we performed differential abundance analysis using DESeq2 (Love et al., 2014) through the DESeq2 Python package, considering taxa with an adjusted *p*-value < 0.05 and absolute log_2_ fold change > 1 as significantly differentially abundant. All statistical analyses were performed in Python 3.8, and plots were generated using Matplotlib and Seaborn (Waskom, 2021).

### 2.9 Plant growth and physiological measurements

Plant height was measured from the base to the flag leaf collar at the silking stage using a measuring tape. A plant canopy analyzer assesses leaf area index (LAI) (Welles and Norman, 1991). Chlorophyll content was determined using a SPAD-502 meter (Uddling et al., 2007). Nitrogen, phosphorus, and potassium content in leaves were determined from samples collected at the tasseling stage. Dried and ground leaf samples were analyzed using the Kjeldahl method for nitrogen and inductively coupled plasma optical emission spectrometry (ICP-OES) for phosphorus and potassium (Jones et al., 1991). Water use efficiency (WUE) was calculated as the ratio of grain yield to total water use during the growing season (Passioura, 2006). Nitrogen use efficiency (NUE) was determined as the ratio of grain yield to total nitrogen applied (Moll et al., 1982). Maize was harvested at physiological maturity, ~120 days after sowing (DAS) (Abendroth et al., 2011). Yield was measured as tons per hectare (tons ha^−1^) by weighing the total biomass of ears per plot after shelling and adjusting to 14% standard moisture content (Zia et al., 2013). The harvest index was calculated as the ratio of grain yield to total aboveground biomass (Hay, 1995; Badu-Apraku et al., 2012). Kernel weight was assessed by weighing 1,000 grains per plot (CIMMYT standard procedures). Cob length and diameter were measured using a digital caliper (Carcova and Otegui, 2001).

Statistical analysis of yield data was performed using one-way ANOVA, followed by Tukey's HSD *post-hoc* test for pairwise comparisons, with significance set at *p* < 0.05. Statistical analyses were performed using R software (version 4.1.0, R Foundation for Statistical Computing, Vienna, Austria) (R Core Team, 2021).

### 2.10 Statistical analysis

All statistical analyses were performed using Python (version 3.9.19) (Van Rossum and Drake, 2009). The following libraries were utilized: pandas (version 2.2.2) for data manipulation and preprocessing (McKinney, 2010), scikit-learn (version 1.5.1) for machine learning-based feature selection (Pedregosa et al., 2011), statsmodels (version 0.14.2) for inferential statistics and hypothesis testing (Seabold and Perktold, 2010), and SciPy (version 1.12.0) for additional statistical computations (Virtanen et al., 2020). Data visualization was performed using matplotlib (version 3.9.2) and seaborn (version 0.13.2) (Hunter, 2007; Waskom et al., 2020).

To assess the relationship between microbial diversity and crop performance, multiple statistical approaches were implemented. The normality of the data was tested using the Shapiro–Wilk test, while Levene's test assessed the homogeneity of variances. When assumptions of parametric tests were met, one-way ANOVA followed by Tukey's HSD *post-hoc* test was applied to compare means across different treatments. For non-parametric comparisons, the Kruskal–Wallis test, followed by Dunn's test with Bonferroni correction, was performed.

Multiple linear regression models were constructed using the ordinary least squares (OLS) method in the statsmodels library to evaluate the influence of microbial diversity and environmental factors (temperature, precipitation, and soil pH) on crop yield and health metrics. The regression model used was:


$$\begin{array}{l} \text{Y}={\text{β}}_{0}+{\text{β}}_{1}{\text{X}}_{1}+{\text{β}}_{2}{\text{X}}_{2}+...+{\text{β}}_{\text{n}}{\text{X}}_{\text{n}}+\text{ε} \end{array}$$

Where *Y* represents the dependent variable (e.g., crop yield or plant health index), X_1_, X_2_,..., X_*n*_ are independent variables (diversity indices, environmental covariates, and fertilization treatment), β_0_, β_1_, β_2_,..., β_*n*_ are regression coefficients, and ε is the error term. Model assumptions were verified using quantile-quantile (Q-Q) plots, the Shapiro–Wilk test for normality of residuals, and the Breusch-Pagan test for homoscedasticity.

Significance levels were set at *p* < 0.05, and 95% confidence intervals were calculated for all parameter estimates. Effect sizes were reported where applicable using Cohen's *f*^2^ for regression models and eta-squared (η^2^) for ANOVA. All statistical analyses were conducted in a fully scripted and reproducible manner, with code available upon request.

## 3 Results

Our study showed that different fertilization strategies significantly affect soil physicochemical properties, microbial community structure, maize yield, and disease incidence. Satellite-derived indices strongly correlate with key soil parameters, validating their use in soil health monitoring. Biofertilization treatments, mainly when applied via drone technology, demonstrated the most pronounced positive impacts on soil microbial diversity, nutrient availability, crop yield, and disease resistance.

### 3.1 Correlation analysis of soil physicochemical parameters and satellite-derived indices

**Figure 1:**
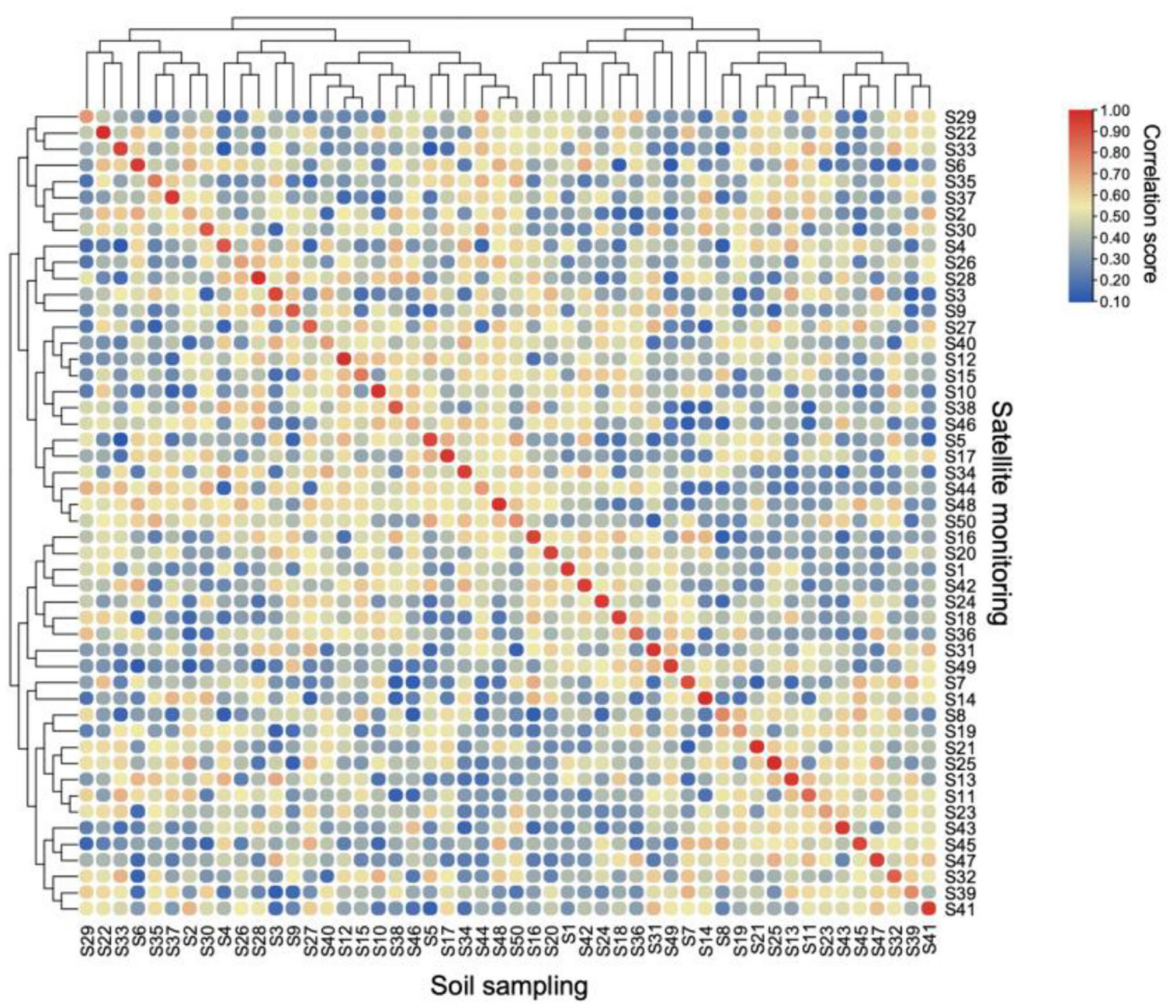
Correlation heatmap comparing soil physicochemical parameters obtained through traditional laboratory analysis with corresponding satellite-derived soil indices (S1–S50, *n* = 50). Colors represent Spearman correlation coefficients ranging from 0.00 (dark blue) to 1.00 (dark red). Hierarchical clustering was performed using Ward's minimum variance method with Euclidean distances. Data were standardized before correlation analysis. Statistical significance was determined using Benjamini-Hochberg adjusted *p*-values (*p* < 0.05).

This analysis showed a remarkably high precision in the correlation between laboratory-measured soil physicochemical properties and spectral indices derived from georeferenced satellite imagery. A highly positive correlation (*r* ≈ 0.98, *p* < 0.001) was evident between laboratory analyses and spectral image analyses for corresponding sample points (e.g., S1 laboratory vs. S1 spectral, S2 laboratory vs. S2 spectral, and so on up to S50) (Figure 1).

Importantly, this high correlation was observed only between matching sample points and not between different field areas (see red diagonal series in Figure 1). For instance, the spectral data for sample S1 showed a near-perfect correlation with the laboratory data for S1 but not with laboratory data from S2, S3, or any other sample points. This pattern was consistent across all 50 sample points (S1–S50).

The strength and specificity of these correlations underscore the accuracy of spectral image analysis in capturing soil physicochemical properties. Critical parameters such as pH, organic matter (OM), total nitrogen (TN), and available phosphorus (AP) all showed correlations of *r* > 0.95 (*p* < 0.001) between laboratory and spectral measurements for each respective sample point.

### 3.2 Impact of fertilization strategies on soil properties, plant physiology, and maize yield

**Figure 2:**
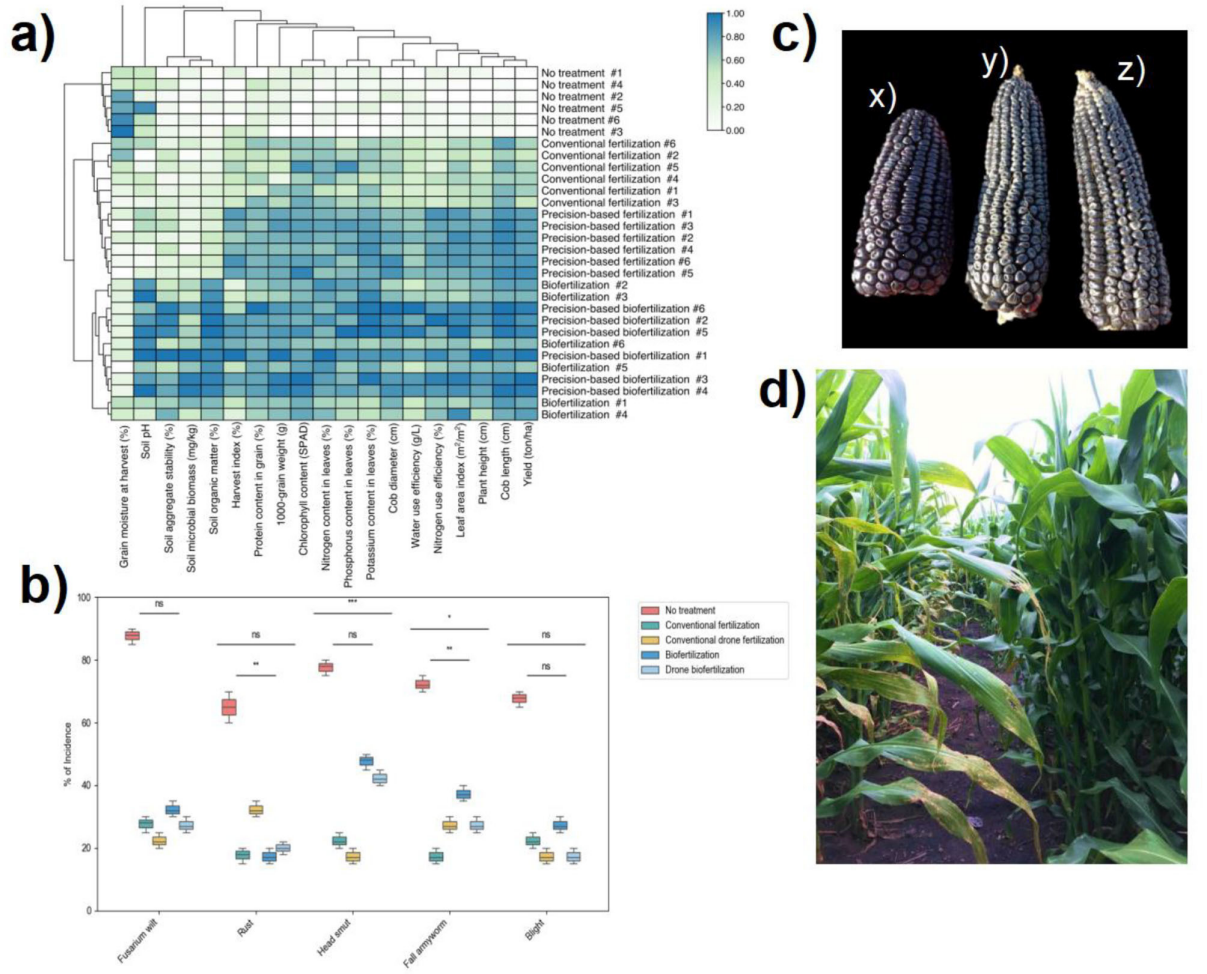
Effects of different fertilization strategies on maize yield and disease incidence. **(a)** Heatmap showing normalized correlations between fertilization treatments, soil properties, and plant performance metrics. Darker shades indicate stronger positive correlations with measured parameters, particularly in biofertilization treatments. **(b)** Box plots showing the distribution of disease incidence (%) for five common maize pathogens under different treatment regimes. **(c)** Representative maize cobs from different treatments: (x) no treatment, (y) conventional drone fertilization, and (z) drone biofertilization. **(d)** Field photograph showing maize plants under control treatment (left) and drone biofertilization treatment (right) at the flowering stage. Statistical significance: *p < 0.05, **p < 0.01, ***p < 0.001; ANOVA followed by Tukey's *post-hoc* test. Error bars represent the standard error of the mean.

The application of different fertilization strategies significantly influenced soil properties, nutrient uptake, plant physiological traits, maize yield, and disease incidence. The heatmap analysis (Figure 2a) showed strong correlations between fertilization treatments and key soil and plant parameters. Biofertilization treatments, particularly drone biofertilization, were associated with higher soil microbial biomass (0.72 ± 0.05), improved soil aggregate stability (0.78 ± 0.03), and increased soil organic matter content (0.86 ± 0.04). In contrast, conventional fertilization resulted in lower microbial biomass (0.46 ± 0.04) and aggregate stability (0.48 ± 0.02), suggesting a reduced impact on soil structure and long-term fertility. Soil pH was significantly higher in biofertilization treatments (6.8 ± 0.1) compared to conventional fertilization (6.3 ± 0.2) and untreated controls (5.9 ± 0.3) [ANOVA, *F*(4, 95) = 21.7, *p* < 0.001].

Biofertilization also enhanced plant nutrient uptake and physiological performance. Nitrogen content in leaves was highest under drone biofertilization (0.86 ± 0.02%), followed by backpack biofertilization (0.80 ± 0.03%) and conventional drone fertilization (0.74 ± 0.02%). In contrast, conventional fertilization and untreated controls had significantly lower nitrogen content (0.63 ± 0.04% and 0.41 ± 0.03%, respectively) (*p* < 0.05). Similar phosphorus and potassium uptake trends were observed, with the highest concentrations detected in biofertilization treatments. Chlorophyll content was also significantly higher in drone biofertilization (48.2 ± 1.8 SPAD) compared to conventional fertilization (42.7 ± 2.1 SPAD) and untreated plants (35.4 ± 2.5 SPAD) (*p* < 0.001), indicating improved photosynthetic capacity.

Plant morphological responses aligned with these physiological changes. Plants in drone biofertilization treatments exhibited greater height (2.1 ± 0.1 m) and larger leaf area index (3.9 ± 0.2) compared to conventionally fertilized (1.8 ± 0.1 m, 3.2 ± 0.1) and untreated plants (1.4 ± 0.2 m, 2.5 ± 0.2) (*p* < 0.05). These growth improvements translated into enhanced cob development, with drone biofertilization resulting in the most considerable cob length (19.4 ± 0.7 cm) and diameter (5.6 ± 0.2 cm), whereas untreated plants produced significantly smaller cobs (11.3 ± 0.9 cm length, 4.1 ± 0.3 cm diameter) (Figure 2c).

Yield outcomes reflected these physiological advantages. Drone biofertilization resulted in the highest mean yield (7.4 ± 0.03 tons/ha), significantly outperforming backpack biofertilization (7.2 ± 0.14 tons/ha), conventional drone fertilization (6.7 ± 0.19 tons/ha), conventional fertilization (6.3 ± 0.33 tons/ha), and untreated controls (2.5 ± 0.21 tons/ha) [ANOVA, *F*(4, 95) = 42.6, *p* < 0.001]. *Post-hoc* Tukey's HSD tests confirmed that drone biofertilization yielded significantly higher than all other treatments (*p* < 0.05) (Figure 2b).

Disease incidence varied significantly across treatments, with biofertilization demonstrating a strong protective effect. Kruskal–Wallis tests, followed by Dunn's *post-hoc* comparisons, showed that drone biofertilization significantly reduced the incidence of Fusarium wilt (20% [IQR: 15–25%]) compared to conventional fertilization (55% [IQR: 50–65%]) and untreated controls (80% [IQR: 70–85%]) (*p* < 0.05). Similar reductions were observed for root rot, stalk rot, and head smut, indicating that biofertilization, particularly when delivered via precision technology, enhances plant resilience against pathogenic infections (Figure 2b).

The visual assessment of cob quality and field performance further supported these quantitative findings. Cobs from drone biofertilization treatments exhibited more uniform kernel filling, greater grain weight, and lower levels of kernel abortion compared to conventionally fertilized and untreated plants (Figure 2c). Additionally, maize plants under biofertilization showed increased vigor, with more robust stems and healthier foliage at the flowering stage compared to the control (Figure 2d).

### 3.3 Taxonomic richness and multivariate analysis of soil microbial communities

**Figure 3:**
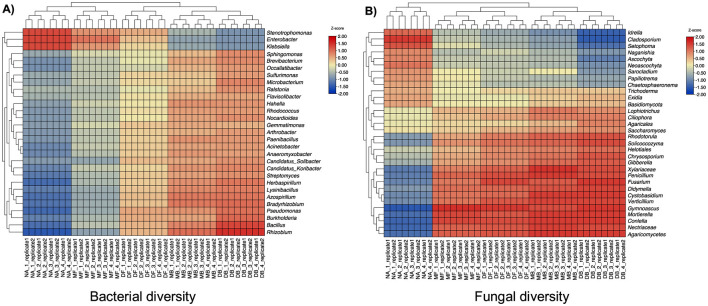
Heatmap visualization of bacterial **(A)** and fungal **(B)** diversity across different treatments. The heatmaps show the relative abundance of different genera, with color intensity representing *Z*-score values ranging from −2.00 (blue) to 2.00 (red). Each column represents a different treatment condition, and taxonomic groups are displayed radially with their corresponding hierarchical clustering dendrograms.

The metabarcoding sequencing of soil samples yielded 2.5 million raw reads across 40 libraries, representing five fertilization treatments: no application, backpack fertilization, drone fertilization, backpack biofertilization, and drone biofertilization, with four sampling points and two replicates each. After quality filtering and chimera removal, 1.8 million high-quality sequences were retained, clustering into 57 distinct genera (27 bacterial and 30 fungal OTUs). Taxonomic classification revealed four major phyla across 14 classes, 25 orders, and 34 families, with Ascomycota (≈65%) and Basidiomycota (≈25%) dominating the fungal community. *Z*-score analysis revealed distinct microbial distribution patterns across treatments, significantly enriching beneficial microorganisms in biofertilization treatments. Control soils (NA) showed a higher abundance of *Cladosporium* (*Z* = 1.55), *Setophoma* (*Z* = 1.33), and *Enterobacter* (*Z* = 1.32), while biofertilized soils, particularly under drone application (DB), exhibited significant enrichment of plant growth-promoting microorganisms including *Rhizobium* (*Z* = 1.51), *Mortierella* (*Z* = 1.53), and *Penicillium* (*Z* = 1.53) (Figure 3A).

Bacterial communities exhibited complementary patterns, with plant growth-promoting rhizobacteria showing significant enrichment in biofertilization treatments. *Rhizobium* abundance increased markedly in DB (*Z* = 1.51) and MB (*Z* = 1.00) treatments, mainly from time points 2 to 4. Similarly, *Pseudomonas* and *Bradyrhizobium* showed progressive enrichment in biofertilization treatments (*Z*-scores increasing from 0.54 to 0.98 and 0.32 to 1.01, respectively). The drone application methods (DB and DF) showed more stable community compositions across replicates than backpack applications (average standard deviation: drone = 0.24, backpack = 0.41). Temporal analysis revealed distinct succession patterns: initial time points (1–2) showed moderate shifts from control conditions (average Δ*Z* = 0.45), while later time points (3–4) exhibited more pronounced community restructuring (average Δ*Z* = 0.89). This temporal progression was particularly evident in biofertilization treatments, where beneficial microorganisms showed consistent enrichment patterns (temporal correlation coefficient *r* = 0.78, *p* < 0.001). *Post-hoc* Dunn's tests with Benjamini-Hochberg correction confirmed that biofertilization treatments, particularly drone-assisted application, resulted in significantly higher microbial richness than other treatments (*p* < 0.05). The most pronounced differences were observed between DB4 and NA1 treatments (average Δ*Z* = 2.14 for fungi, Δ*Z* = 1.89 for bacteria). The progressive increase in *Z*-scores from NA to DB treatments, particularly evident in time points 3 and 4, suggests a cumulative positive effect of biofertilization on microbial community structure (Figure 3B).

**Figure 4:**
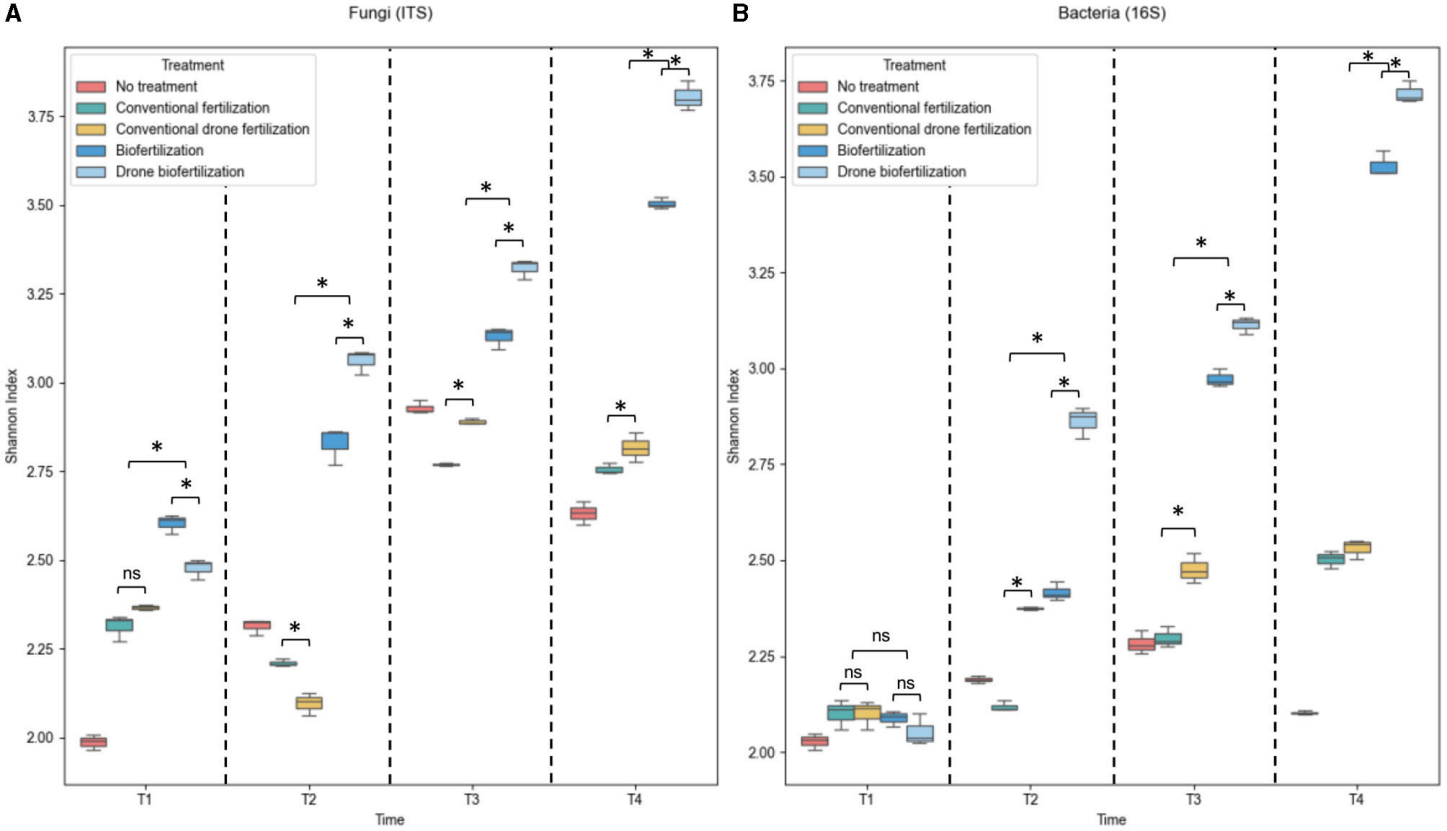
Taxonomic richness of soil microbial communities under different fertilization treatments. **(A)** Box plots showing fungal (ITS region) diversity across treatments at different time points. **(B)** Box plots showing bacterial (16S rRNA) diversity across treatments at different time points. Boxes represent interquartile ranges (IQR), horizontal lines indicate medians, whiskers extend to 1.5 × IQR, and points show outliers. Asterisks indicate significant differences between treatments (*p < 0.05), ns = not significant.

Bacterial and fungal communities analysis revealed significant differences in taxonomic composition and abundance patterns across treatments and time points (Figure 4). Kruskal–Wallis tests showed substantial variations among treatments for both fungi [χ^2^(4) = 38.2, p < 0.001] and bacteria [χ^2^(4) = 42.7, *p* < 0.001]. For fungi, drone biofertilization (DB) showed the highest enrichment in beneficial genera, with *Mortierella* (*Z* = 1.53), *Penicillium* (*Z* = 1.53), and *Fusarium* (*Z* = 1.56) increasing significantly from time points 1 to 4. Backpack biofertilization (MB) showed similar but less pronounced trends (average *Z*-scores rising from 0.68 to 1.21 across time points). In contrast, chemical fertilization treatments (MF and DF) showed moderate enrichment (*Z*-scores ranging from 0.29 to 0.84), with drone application (DF) showing more consistent patterns than backpack application (MF) (coefficient of variation: DF = 18.2%, MF = 27.4%).

**Figure 5:**
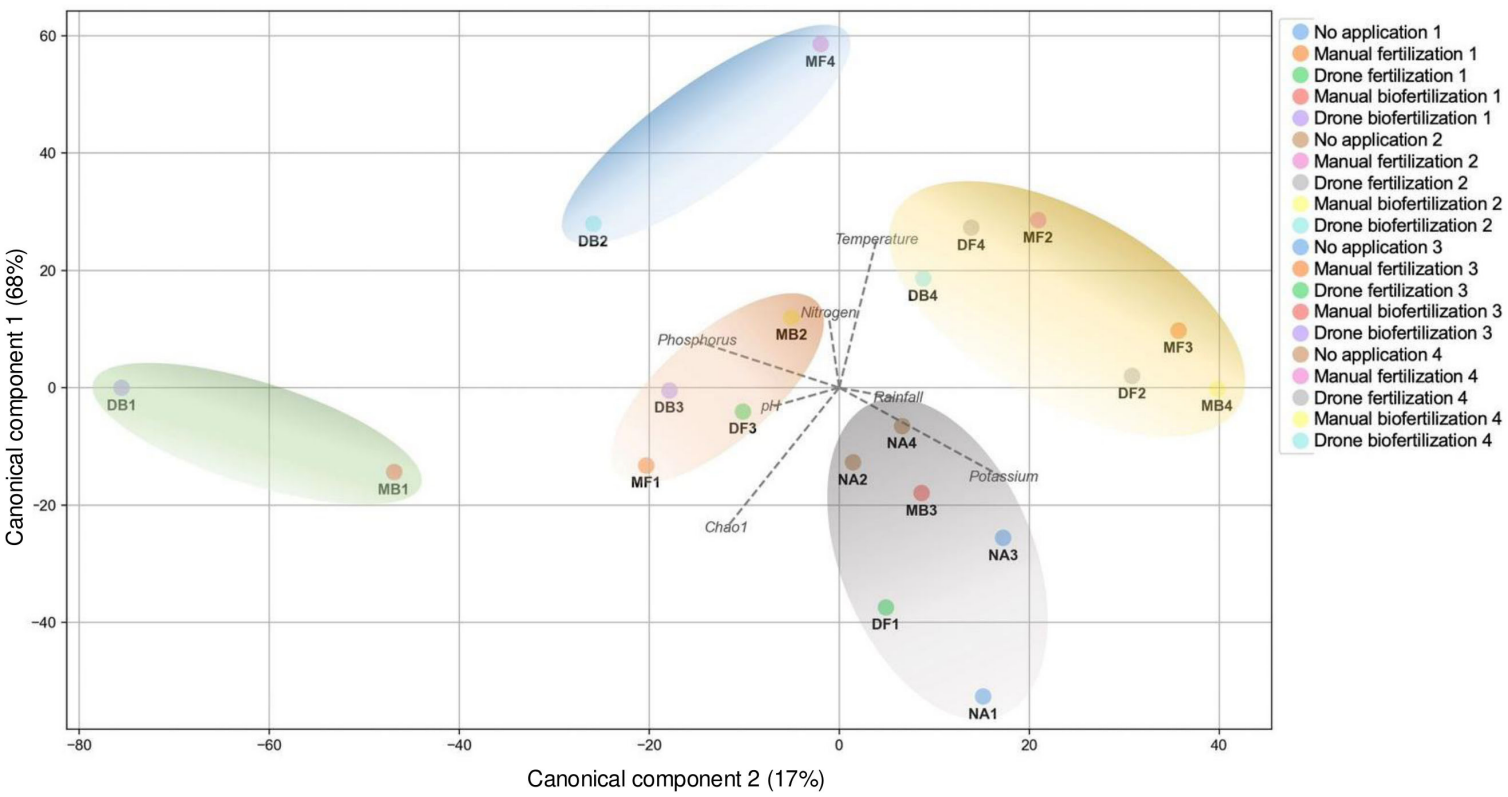
Canonical Correlation Analysis (CCA) of treatment groupings based on environmental variables. Vectors represent correlations between ordination axes and soil parameters: microbial richness, pH, temperature, and nutrient availability (N, P, K, measured in mg/kg). Colored ellipses represent 95% confidence intervals for treatment groups. The first and second canonical axes explain X% and Y% of the total variance, respectively. Data were standardized before analysis. The significance of the canonical correlations was tested using permutation tests (999 permutations, *p* < 0.05).

Canonical Correlation Analysis (CCA) of treatment groupings based on environmental variables (Figure 5) revealed that the first two canonical axes explained 68 and 17% of the total variance, respectively. Permutation tests (999 permutations) confirmed the significance of the canonical correlations (*p* < 0.001). Microbial diversity and availability of nutrients, particularly nitrogen (N) and phosphorus (P), were positively associated with biofertilization. The CCA plot shows a clear separation of treatment groups, with drone biofertilization (DB) clustering distinctly from other treatments and associated with higher nutrient availability.

**Figure 6:**
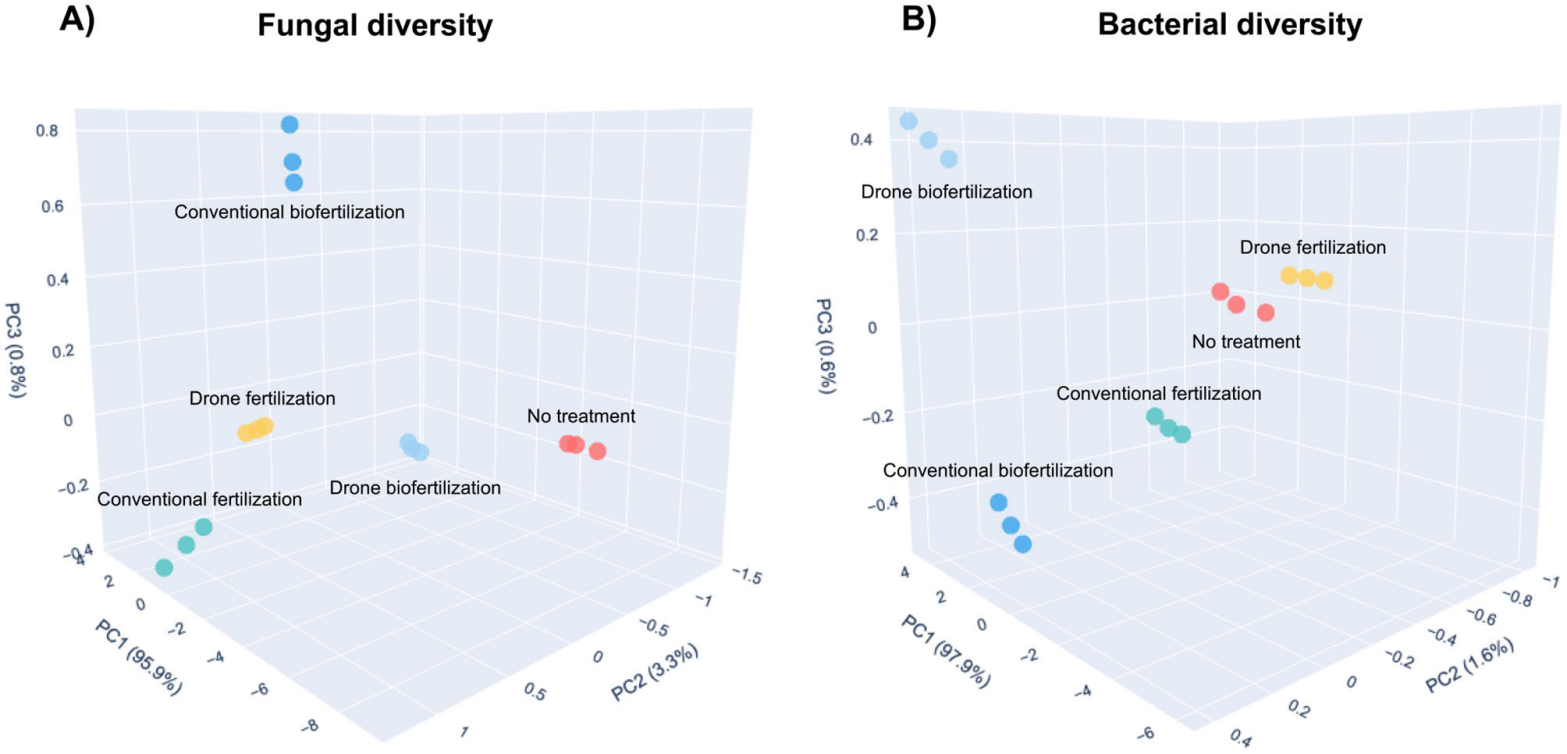
Three-dimensional Principal Coordinate Analysis (PCoA) of weighted UniFrac distances shows soil microbial communities' beta diversity patterns under different fertilization treatments. **(A)** Fungal community structure separates treatment groups, with PC1, PC2, and PC3 explaining 95.9%, 3.3%, and 0.8% of the total variation, respectively. **(B)** Bacterial community structure depicting distinct clustering patterns among treatments, with PC1, PC2, and PC3 explaining 97.9%, 1.6%, and 0.6% of the total variation, respectively. Treatments are color-coded: No treatment (red), Conventional fertilization (turquoise), Conventional drone fertilization (yellow), Biofertilization (blue), and Drone biofertilization (light blue). The percentage of variation explained by each principal coordinate is shown in parentheses on the respective axes.

The weighted UniFrac Principal Coordinate Analysis (PCoA) revealed distinct clustering patterns for fungal and bacterial communities across different fertilization treatments. For fungal communities (Figure 6A), the first three principal coordinates explained 99.9% of the total variation (PC1: 95.9%, PC2: 3.3%, and PC3: 0.8%). The no-treatment control was separated from the fertilization treatments, with biofertilization treatments (both drone and conventional applications) forming a distinct cluster. Conventional fertilization and drone fertilization treatments showed intermediate positioning, suggesting a gradual shift in community composition.

The bacterial community structure (Figure 6B) showed a different pattern, with the three principal coordinates explaining 99.8% of the total variation (PC1: 97.6%, PC2: 1.6%, and PC3: 0.6%). The treatments exhibited clear spatial separation, with drone biofertilization and conventional drone fertilization clustering distinctly from conventional fertilization and no-treatment controls. Notably, the biofertilization treatment showed an intermediate position between conventional and drone-based applications, suggesting a gradient of community composition changes influenced by the fertilizer and application methods.

### 3.4 Microbial network dynamics in response to drone-assisted biofertilization

**Figure 7:**
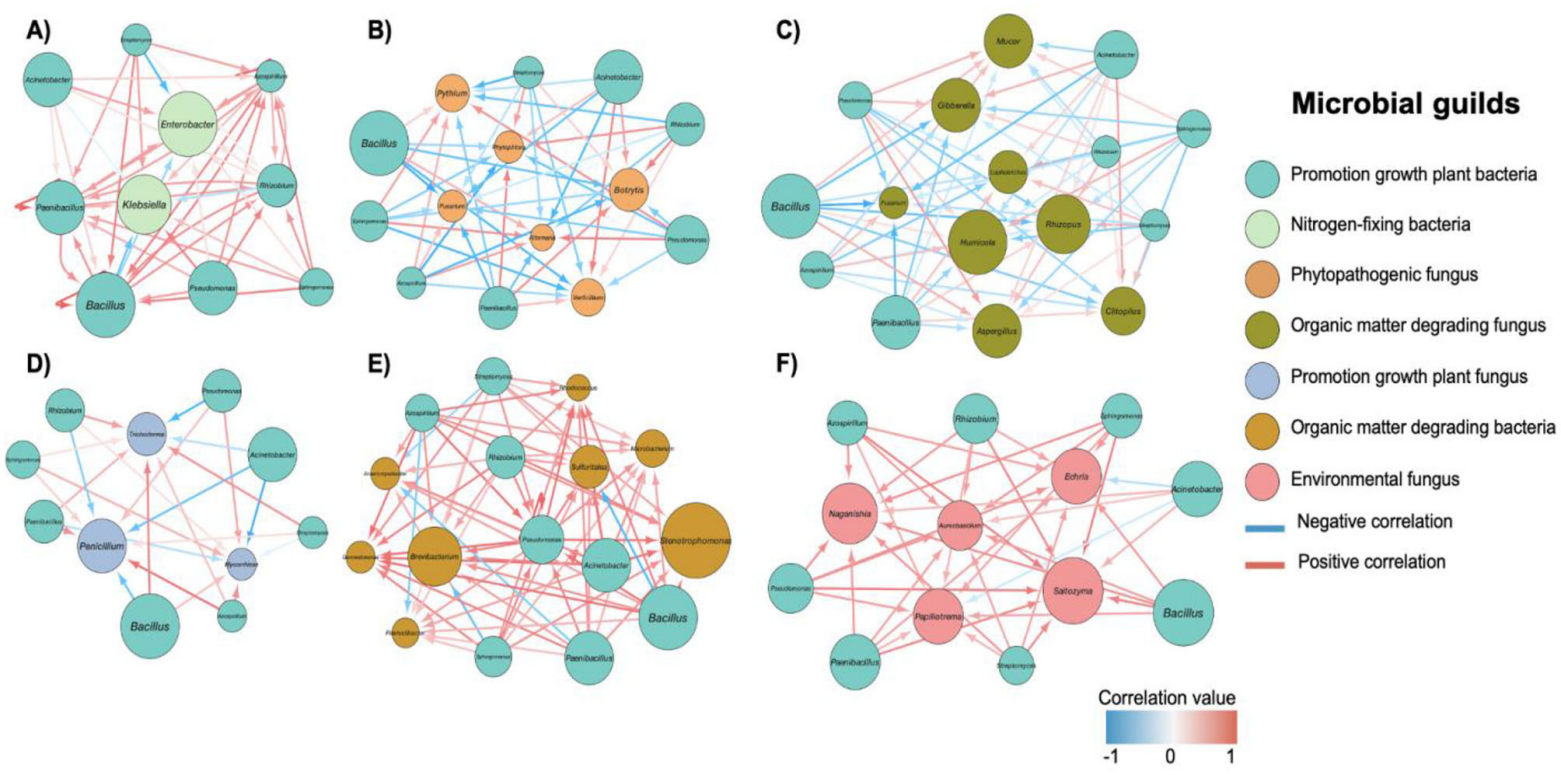
Network analysis of microbial interactions under different fertilization regimes. **(A)** Control (no fertilization); **(B)** Conventional fertilization; **(C)** Biofertilization; **(D)** Drone-assisted conventional fertilization; **(E)** Drone-assisted biofertilization; **(F)** Environmental fungi interactions. Node colors represent different microbial guilds, as indicated in the legend. Node size is proportional to the relative abundance of each taxon. Edge colors indicate positive (red) or negative (blue) correlations, with intensity proportional to the strength of the correlation. Only significant correlations (*p* < 0.05) with an absolute value > 0.6 are shown. PGPB, Plant Growth-Promoting Bacteria; PGPF, Plant Growth-Promoting Fungi.

Network analysis revealed complex interactions among microbial guilds in response to drone-assisted biofertilization based on correlated changes in abundance throughout the crop cycle (Figure 7). Bacteria with potential plant growth-promoting abilities showed strong positive correlations among themselves (*r* > 0.8), particularly between *Bacillus, Pseudomonas*, and *Acinetobacter* suggesting a synergistic relationship in their response to biofertilization (Figure 7A). Potential nitrogen-fixing bacteria, represented by *Enterobacter* and *Klebsiella*, displayed positive correlations (*r* ≈ 0.6 to 0.8) with several PGPB (Figure 7A), suggesting a cooperative response to the treatment.

Interestingly, phytopathogenic fungi showed varied responses (Figure 7B). While some, like *Pythium*, exhibited negative correlations with PGPB (*r* ≈ −0.7), others, like *Botrytis*, showed weaker negative correlations (*r* ≈ −0.4), suggesting differential suppression. Organic matter-degrading fungi, including *Mucor* and *Rhizopus*, demonstrated positive correlations (*r* > 0.7) with both PGPB and nitrogen-fixing bacteria, indicating facilitation of nutrient cycling by these fungi in response to biofertilization (Figure 7). Plant growth-promoting fungi, particularly *Penicillium* and *Trichoderma*, showed strong positive correlations (*r* > 0.8) with PGPB (Figure 7D), suggesting a complementary response to the treatment. The network involving organic matter-degrading bacteria (Figure 7E) suggests a strong positive correlation (*r* > 0.8) with PGPB and nitrogen-fixing bacteria, indicating an integrated response in nutrient mobilization and plant growth promotion. Environmental fungi displayed varied correlations with other microbial groups (Figure 7F). Some genera, like *Naganishia*, showed moderate positive correlations (*r* ≈ 0.5 to 0.7) with PGPB, while others exhibited weak to moderate negative correlations (*r* ≈ −0.3 to −0.6), suggesting diverse ecological roles in the biofertilized soil.

These results highlight the complex, interconnected response of the soil microbiome to drone-assisted biofertilization and reveal potential synergies and antagonisms that emerged throughout the treatment.

## 4 Discussion

The structure of the soil microbial community was analyzed under various agricultural treatments, revealing substantial shifts in bacterial composition associated with fertilization strategies. The current study demonstrates that the number of applications and the type of treatment—whether backpack, drone-assisted, or biofertilizer—significantly affected the relative abundances of dominant and subdominant bacterial taxa.

Our analysis of untreated agricultural soils indicated a marked predominance of Enterobacteriaceae, with *Enterobacter* and *Klebsiella* genera constituting a significant portion of the bacterial community. This aligns with Köberl et al. (2011) finding that Enterobacteriaceae is a dominant family in agricultural soils from Egypt (25% of total sequences), particularly in non-organic farming systems. Chen et al. (2019) also observed this dominance pattern in conventional rice cropping systems, where Enterobacteriaceae members significantly increased under conventional fertilization practices. These observations are consistent with the work of Fierer et al. (2007), who demonstrated the capacity of these genera to thrive under nutrient-depleted conditions. The prevalence of such opportunistic bacteria typically indicates disturbed or simplified ecosystems, as Van Elsas et al. (2012) documented in their comprehensive study of soil microbial dynamics.

The microbial shifts observed in this study following the application of teosinte-derived SynCom and precision biofertilization suggest that targeted microbial inoculation can lead to functionally enriched soil communities. The significant reduction in *Enterobacteriaceae*, coupled with the increase in *Bacillus, Pantoea*, and *Rhizobiales*, aligns with previous research demonstrating that biofertilization promotes beneficial microbial groups that enhance nutrient cycling and plant resistance to stress (Bargaz et al., 2018; Deng et al., 2019a,b). Similar patterns have been reported in soil microbiome studies where the introduction of plant growth-promoting bacteria (*PGPB*) increases microbial diversity and network connectivity (Zhalnina et al., 2018). Additionally, the observed increase in *Verrucomicrobia* and *Acidobacteria*, often associated with stable and resilient soil environments, suggests that biofertilization not only enhances microbial diversity but also contributes to long-term soil functionality (Fierer et al., 2007).

Additionally, we observed a significant presence of Firmicutes, predominantly *Bacillus* species (Mandic-Mulec et al., 2015), consistent with the findings correspond with previous studies highlighting the adaptability of spore-forming bacteria to environments with variable nutrient availability (Lennon and Jones, 2011; Shade et al., 2012). Such limited microbial diversity is often a hallmark of agricultural soils subject to minimal management, as noted in recent meta-analyses of soil microbiome structures (Delgado-Baquerizo et al., 2020).

The conventional backpack fertilization induced a significant shift in microbial composition, with the relative abundance of *Enterobacter* and *Klebsiella* decreasing over successive applications (Geisseler and Scow, 2014). Conversely, genera such as *Pseudomonas* and *Lysinibacillus* exhibited an increase in relative abundance (Zhalnina et al., 2018). *Pseudomonas*, renowned for their metabolic versatility and plant growth-promoting attributes, appears to capitalize on the enhanced nutrient availability of fertilization (Paungfoo-Lonhienne et al., 2015). The proliferation of *Lysinibacillus*, a genus within the Firmicutes phylum, suggests that fertilization supports the growth of selected taxa and facilitates the emergence of new microbial competitors. *Lysinibacillus* is known for its significant contributions to soil health, particularly in the decomposition of organic matter and nutrient cycling processes, both contributors to overall soil fertility (Ding et al., 2013; Caulier et al., 2019). These alterations in microbial community structure underscore the complex ecological dynamics triggered by conventional fertilization practices in agricultural soils.

Four applications of drone-assisted fertilization yielded a similar trend to backpack methods but with more pronounced impacts on microbial diversity (Adak et al., 2012). The dominance of *Enterobacter* and *Klebsiella* decreased with consecutive applications, mirroring the trend observed in backpack fertilization (Kavamura et al., 2018). Notably, other bacterial genera, including *Bacillus, Pseudomonas*, and *Rhizobium*, became significant microbial community members under these conditions (Zhang et al., 2015). Those genera are highly adaptable and can thrive in various environmental conditions, including those with elevated levels of anthropogenic inputs (Lori et al., 2023). Many of those genera are known for their diverse metabolic capabilities, including the degradation of complex organic compounds that may contribute to enhancing nutrient cycling in fertilized soils (Leff et al., 2015).

The proliferation of various bacterial groups, particularly those involved in nutrient cycling, further emphasizes the impact of drone-assisted fertilization on microbial dynamics (Zheng et al., 2019). The seemingly increased abundances of specific genera, such as *Rhizobium, Bacillus, Pseudomonas, Azospirillum, Burkholderia, Bradyrhizobium*, and *Streptomyces*, crucial contributors to nitrogen fixation, phosphate solubilization, and overall soil health enhancers, suggests that this fertilization method may enhance soil nutrient levels and promote the growth of beneficial bacteria (Gao et al., 2019). Such shifts could yield long-term benefits for soil health and plant productivity, fostering a more sustainable agricultural ecosystem (Bender et al., 2016).

The more pronounced alterations observed in drone-assisted fertilization compared to backpack methods may be attributed to the enhanced precision and uniformity of application achieved with drone technology (Mogili and Deepak, 2018). This precision potentially results in a more homogeneous distribution of nutrients, creating microenvironments that favor the proliferation of specific bacterial genera, such as *Bacillus, Pseudomonas, Rhizobium, Burkholderia, Azospirillum, Bradyrhizobium*, and *Lysinibacillus*. Furthermore, drone-assisted fertilization does not contribute to soil compaction compared to backpack or machine-based fertilization, which may contribute to a soil structure that is more favorable to microbial communities (Cardoso et al., 2013).

Biofertilization, whether backpack or drone-assisted, induced the most significant changes in microbial community structure, characterized by a marked increase in diversity (Bargaz et al., 2018). The relative abundance of specific bacterial genera decreases (e.g., *Enterobacter, Stenotrophomonas*, and *Klebsiella*), while others, such as *Burkholderia* and *Azospirillum*, become more prominent (Kour et al., 2020). *Burkholderia* species are known for their biosynthetic potential to produce complex organic compounds that contribute to plant growth through various mechanisms, including nitrogen fixation and plant growth-promoting hormones (Suárez-Moreno et al., 2012). The increase in *Azospirillum*, a well-established genus known for its nitrogen-fixing capabilities and association with the rhizosphere, suggests that biofertilization not only enriches the soil microbiome but also specifically enhances the populations of bacteria that directly benefit plant health (Bashan and de-Bashan, 2010; Fukami et al., 2018).

Biofertilization treatments also increased Verrucomicrobia and Acidobacteria, which include species like *Solibacter, Koribacter*, and *Occallatibater*, often associated with healthy, stable soils (Fierer et al., 2007). The increased presence of Verrucomicrobia, known for their roles in carbon cycling and environmental resilience, indicates a shift toward a more functionally diverse and potentially more resilient soil ecosystem (Bergmann et al., 2011). Acidobacteria are recognized for their adaptability to varied soil conditions and ability to degrade complex organic materials, further contributing to the overall health and sustainability of the soil environment (Kielak et al., 2016).

The comparative analysis of backpack and drone-assisted applications suggests that drone-assisted biofertilization may yield a more uniform distribution of beneficial microbes, leading to a more substantial increase in microbial diversity (Deng et al., 2019a,b). However, it is essential to consider the potential long-term implications of these changes. While increased microbial diversity is generally associated with enhanced soil health and resilience (Wagg et al., 2014), introducing specific genera at high abundance could lead to microbial community structure and functionality shifts. These changes must be monitored to ensure they align with sustainable agricultural practices and optimal nutrient cycling in the soil (Vitousek et al., 2013; Kuypers et al., 2018).

In untreated soils, fungi genera such as *Cladosporium, Nigrospora*, and *Sordaria* are relatively more abundant (Tedersoo et al., 2014). *Cladosporium* is known for its resilience in various environments, including soils, where it acts as a saprotroph, decomposing organic matter (Bensch et al., 2010). *Nigrospora* and *Sordaria* are similarly adapted to less disturbed soils, often associated with decomposing plant material (Ma et al., 2022). The presence of Ascomycota in untreated soils suggests a fungal community dominated by decomposers, reflecting a stable ecosystem with balanced organic matter turnover (Sterkenburg et al., 2015).

The introduction of conventional backpack fertilization leads to a notable increase in the relative abundance of specific fungal genera (Francioli et al., 2016). Some *Fusarium* species, potential plant pathogens, show increased presence, potentially indicating a risk for crops under these fertilization regimes (Moretti et al., 2017). *Aspergillus*, another genus that thrives in nutrient-rich environments, also becomes more prominent, possibly benefiting from increased nutrient availability (Houbraken et al., 2014).

Drone-assisted conventional fertilization exhibits a similar pattern but with an even higher relative abundance of specific fungal genera as the number of applications increases (Zhu et al., 2016). Specifically, genera such as *Fusarium, Penicillium, Verticillium, Gibberella*, and *Chrysosporium* show a marked increase in relative abundance under drone-assisted fertilization conditions. This suggests that drone-assisted fertilization might create conditions favoring the proliferation of these genera, potentially leading to an increased risk of soil-borne diseases. The presence of lignin-degrading fungi in both backpack and drone-assisted treatments indicates an active breakdown of lignin-rich organic matter, which could be a response to increased organic inputs from fertilization (Osono and Takeda, 2006).

Backpack and drone-assisted biofertilization treatments exhibit the most significant changes in the fungal community. These treatments are characterized by a substantial increase in the relative abundance of *Mortierella, Penicillium*, and *Trichoderma*, genera known for their beneficial effects on soil health and plant growth (Frac et al., 2018). *Mortierella* is often associated with improved soil structure and nutrient cycling (Li et al., 2020), while *Penicillium* and *Trichoderma* are well-known for their roles in biocontrol, suppressing soil-borne pathogens, and promoting plant health (Harman et al., 2004).

In addition to the beneficial taxa, Agaricomycetes are observed in biofertilization treatments. These genera are typically found in forest soils and are known for their mycorrhizal associations, which enhance plants' nutrient uptake (Tedersoo and Smith, 2013). Their presence in agricultural soils suggests biofertilization may foster a soil environment more conducive to mutualistic relationships, potentially leading to enhanced plant growth and resilience (van der Heijden et al., 2015). The data also reveal significant increases in other beneficial fungal genera such as *Trichoderma*, known for its biocontrol properties, and *Mortierella*, associated with phosphate solubilization. The clustering patterns are compared to backpack and drone-assisted biofertilization, which leads to a more homogeneous distribution of fungal taxa. The relative abundance of potentially pathogenic fungi is reduced in biofertilization treatments compared to conventional fertilization, suggesting a shift toward a community dominated by beneficial fungi (Berendsen et al., 2012).

The increased microbial diversity, particularly in biofertilization treatments, may improve soil health through enhanced nutrient cycling, disease suppression, and promotion of beneficial plant-microbe interactions (Trivedi et al., 2020). However, the high abundance of specific genera in biofertilization treatments raises questions about potential long-term effects on soil microbial balance and function (Dubey et al., 2019).

Across all treatments, *Bacillus* emerges as a central node within the microbial networks, consistently showing strong positive correlations with other beneficial bacteria and fungi (Viswanath et al., 2021). *Bacillus* is well-known for its plant growth-promoting properties, including the production of antibiotics, enzymes, and phytohormones that enhance plant health and protect against pathogens (Radhakrishnan et al., 2017). Its central role across different treatments suggests that it is a keystone species in these soil ecosystems, critical for maintaining a healthy microbial balance (Banerjee et al., 2018). As agricultural inputs are applied—whether through conventional or biofertilization methods—there is a noticeable increase in network complexity, characterized by a more significant number of positive and negative interactions between various microbial taxa (Mahanty et al., 2017). The introduction of fertilization generally enhances the presence of nutrient cycling and plant growth-promoting bacteria, which are crucial for nitrogen fixation and overall soil fertility. However, this also coincides with an increased presence of potentially pathogenic microorganisms, particularly under conventional fertilization (Rousk and Bååth, 2011). These organisms tend to thrive in nutrient-rich environments, suggesting that while fertilization promotes beneficial microbial activity, it also creates conditions that could facilitate the proliferation of soil-borne diseases.

Biofertilization, mainly when applied with drone technology, significantly alters the microbial community structure, leading to more intricate and potentially beneficial networks (Deng et al., 2019a,b). The rise of beneficial fungi *Trichoderma, Penicillium*, and *Mortierella*, known for their biocontrol properties, indicates that biofertilization fosters a more resilient soil ecosystem (Keswani et al., 2019). These fungi form positive correlations with key bacterial genera, suggesting a synergistic effect that could enhance soil health and plant growth. The mutualistic relationships, particularly involving mycorrhizal fungi, further support the idea that biofertilization promotes a balanced and functionally diverse soil microbiome (Emam, 2016).

The networks under biofertilization treatments are notable for the emergence of genera involved in organic matter degradation and nutrient cycling (Jacoby et al., 2017). As beneficial bacteria like *Rhizobium* and *Bacillus* increase abundance through successive biofertilization applications, opportunistic fungi like *Cladosporium* and *Setophoma* significantly decrease. Similarly, while nitrogen-fixing bacteria such as *Bradyrhizobium* and *Azospirillum* increase their presence, potentially pathogenic fungi like *Ascochyta* and *Neoascochyta* decline. Conversely, as beneficial fungi like *Trichoderma* and *Penicillium* establish themselves, opportunistic bacteria such as *Enterobacter* and *Stenotrophomonas* show marked reductions. These negative correlations suggest a competitive exclusion process where beneficial microorganisms may actively suppress potentially pathogenic or opportunistic species, contributing to a more balanced and healthy soil ecosystem. The increased complexity of the microbial interactions in these treatments suggests that biofertilization supports plant health and contributes to long-term soil sustainability by promoting processes that recycle organic matter and improve soil fertility (Lehmann et al., 2017).

While biofertilization enhances beneficial interactions within the microbial community, increasing complexity can lead to challenges in microbial management (Parnell et al., 2016). The more complex the network, the greater the potential for beneficial and antagonistic interactions. For example, certain environmental microorganisms can negatively correlate with plant growth-promoting bacteria, suggesting that not all interactions are valuable and that introducing biofertilizers must be carefully managed to avoid unintended consequences (Chaparro et al., 2012).

The antagonistic relationships between certain fungi and bacteria highlight the need for a nuanced approach to fertilization strategies (Mendes et al., 2011). While promoting beneficial microbes is desirable, monitoring and managing the soil environment is crucial for preventing the dominance of potentially harmful species. The balance between promoting plant growth and controlling pathogens is delicate, and the success of biofertilization treatments may hinge on maintaining this balance (Raaijmakers and Mazzola, 2016).

In summary, our study reveals the intricate dynamics of soil microbial communities in response to various fertilization strategies. The transition from conventional methods to biofertilization, mainly when applied via drone technology, demonstrates significant shifts in microbial community composition. We observed the decline of potentially pathogenic genera, such as *Cladosporium* and *Setophoma*, concurrent with increased beneficial bacteria, including *Bacillus* and *Rhizobium*. These changes were accompanied by establishing beneficial fungi like *Trichoderma* and *Mortierella*, while opportunistic bacteria such as *Enterobacter* showed marked reductions. However, it is essential to recognize that these microbial community structure shifts present opportunities and challenges. While the increased abundance of plant growth-promoting microorganisms and nitrogen-fixing bacteria (e.g., *Bradyrhizobium, Azospirillum*) suggests enhanced soil functionality, the persistent presence of some potentially pathogenic fungi (e.g., *Fusarium*) requires careful monitoring. The emergence of *Bacillus* as a keystone species across treatments and the development of complex bacterial-fungal networks involving *Penicillium* and Agaricomycetes underscores the potential for targeted microbial management in agricultural systems. These temporal dynamics and microbial interactions suggest biofertilization, mainly when delivered through drone technology, may promote a more balanced and resilient soil ecosystem. However, maintaining this equilibrium will require informed management strategies.

## 5 Conclusion

This study provides valuable insights into how soil microbial communities respond to fertilization strategies, highlighting microbial succession patterns and community restructuring. Our findings demonstrate that conventional fertilization and biofertilization treatments induce distinct shifts in the soil microbiome. Notably, biofertilization applications, particularly when delivered via drone technology, were associated with an increased abundance of beneficial microorganisms such as *Bacillus* and *Rhizobium*, while the presence of potentially pathogenic organisms decreased over time. The temporal analysis of bacterial-fungal networks suggests that successive biofertilization applications drive significant microbial restructuring, though the long-term stability of these changes warrants further investigation. Understanding these dynamics is crucial for refining biofertilizer formulations and application strategies to maximize their benefits for soil health and crop productivity. Beyond identifying microbiome shifts, this study reinforces the role of biofertilizers as a viable tool for sustainable agriculture. By demonstrating their ability to enhance beneficial microbial communities while suppressing harmful taxa, our findings contribute to advancing biofertilizer-based management strategies that improve soil functionality and resilience. Future research should focus on elucidating the mechanistic pathways underlying these microbial interactions and assessing their agronomic impacts over multiple growing seasons. These insights pave the way for more effective and ecologically sound fertilization practices that integrate soil microbiome management, ensuring long-term agricultural sustainability while maintaining high productivity.

## Data Availability

The datasets presented in this study can be found in Figshare online repositories. The link to access is https://figshare.com/s/0e2c364b0f90d06e67b5.

## References

[B1] Abendroth L. J. Elmore R. W. Boyer M. J. Marlay S. K. (2011). Corn Growth and Development. PMR 1009. Ames, IA: Iowa State University Extension and Outreach.

[B2] Adak T. Kumar J. Shakil N. A. Walia S. (2012). Development of controlled release formulations of imidacloprid employing novel nano-ranged amphiphilic polymers. J. Environ. Sci. Health B. 47, 217–225. 10.1080/03601234.2012.634365 22375594

[B3] Assmann J. J. Kerby J. T. Cunliffe A. M. Myers-Smith I. H. (2019). Vegetation monitoring using multispectral sensors—best practices and lessons learned from high latitudes. J. Unmanned Veh. Syst. 7, 54–75. 10.1139/juvs-2018-0018

[B4] Badu-Apraku B. Fakorede M. A. B. Menkir A. Sanogo D. (2012). Conduct and Management of Maize Field Trials. Ibadan: IITA.

[B5] Bamdad H. Papari S. Lazarovits G. Berruti F. (2022). Soil amendments for sustainable agriculture: microbial organic fertilizers. Soil Use Manag. 38, 94–120. 10.1111/sum.12762

[B6] Banerjee S. Schlaeppi K. van der Heijden M. G. A. (2018). Keystone taxa as drivers of microbiome structure and functioning. Nat. Rev. Microbiol. 16, 567–576. 10.1038/s41579-018-0024-1 29789680

[B7] Bao S. D. (2000). Soil and Agricultural Chemistry Analysis, 3rd Edn. Beijing: China Agriculture Press.

[B8] Bargaz A. Lyamlouli K. Chtouki M. Zeroual Y. Dhiba D. (2018). Soil microbial resources for improving fertilizers efficiency in an integrated plant nutrient management system. Front. Microbiol. 9:1606. 10.3389/fmicb.2018.01606 30108553 PMC6079243

[B9] Bashan Y. de-Bashan L. E. (2010). How the plant growth-promoting bacterium *azospirillum* promotes plant growth—a critical assessment. Adv. Agron. 108, 77–136. 10.1016/S0065-2113(10)08002-8

[B10] Bashan Y. de-Bashan L. E. Prabhu S. R. Hernandez J.-P. (2014). Advances in plant growth-promoting bacterial inoculant technology: formulations and practical perspectives (1998–2013). Plant Soil 378, 1–33. 10.1007/s11104-013-1956-x

[B11] Bellon Maurel V. Huyghe C. (2017). Putting agricultural equipment and digital technologies at the cutting edge of agroecology. OCL 24:D307. 10.1051/ocl/2017028

[B12] Bender S. F. Wagg C. van der Heijden M. G. A. (2016). An underground revolution: biodiversity and soil ecological engineering for agricultural sustainability. Trends Ecol. Evol. 31, 440–452. 10.1016/j.tree.2016.02.016 26993667

[B13] Bensch K. Groenewald J. Z. Dijksterhuis J. Starink-Willemse M. Andersen B. Summerell B. A.. (2010). Species and ecological diversity within the *cladosporium cladosporioides* complex (*davidiellaceae, capnodiales*). *Stud. Mycol*. 67, 1–94. 10.3114/sim.2010.67.01 20877444 PMC2945380

[B14] Berendsen R. L. Pieterse C. M. Bakker P. A. (2012). The rhizosphere microbiome and plant health. Trends Plant Sci. 17, 478–486. 10.1016/j.tplants.2012.04.001 22564542

[B15] Bergmann G. T. Bates S. T. Eilers K. G. Lauber C. L. Caporaso J. G. Walters W. A.. (2011). The under-recognized dominance of verrucomicrobia in soil bacterial communities. Soil Biol. Biochem. 43, 1450–1455. 10.1016/j.soilbio.2011.03.012 22267877 PMC3260529

[B16] Bhardwaj D. Ansari M. W. Sahoo R. K. Tuteja N. (2014). Biofertilizers function as key player in sustainable agriculture by improving soil fertility, plant tolerance and crop productivity. Microb. Cell Fact. 13:66. 10.1186/1475-2859-13-66 24885352 PMC4022417

[B17] Bhattacharyya P. N. Jha D. K. (2012). Plant growth-promoting rhizobacteria (PGPR): emergence in agriculture. World J. Microbiol. Biotechnol. 28, 1327–1350. 10.1007/s11274-011-0979-9 22805914

[B18] Bolyen E. Rideout J. R. Dillon M. R. Bokulich N. A. Abnet C. C. Al-Ghalith G. A.. (2019). Reproducible, interactive, scalable and extensible microbiome data science using QIIME 2. Nat. Biotechnol. 37, 852–857. 10.1038/s41587-019-0209-9 31341288 PMC7015180

[B19] Bray J. R. Curtis J. T. (1957). An ordination of the upland forest communities of Southern Wisconsin. Ecol. Monogr. 27, 325–349. 10.2307/1942268

[B20] Callahan B. J. McMurdie P. J. Rosen M. J. Han A. W. Johnson A. J. A. Holmes S. P. (2016). DADA2: high-resolution sample inference from illumina amplicon data. Nat. Methods 13, 581–583. 10.1038/nmeth.3869 27214047 PMC4927377

[B21] Caporaso J. G. Lauber C. L. Walters W. A. Berg-Lyons D. Lozupone C. A. Turnbaugh P. J.. (2011). Global patterns of 16S rRNA diversity at a depth of millions of sequences per sample. Proc. Natl. Acad. Sci. U.S.A. 108, 4516–4522. 10.1073/pnas.1000080107 20534432 PMC3063599

[B22] Carcova J. Otegui M. E. (2001). Ear temperature and pollination timing effects on maize kernel set. Crop Sci. 41, 1809–1815. 10.2135/cropsci2001.1809

[B23] Cardoso E. J. B. N. Vasconcellos R. L. F. Bini D. Miyauchi M. Y. H. Santos C. A. Alves P. R. L.. (2013). Soil health: looking for suitable indicators. What should be considered to assess the effects of use and management on soil health? Sci. Agric. 70, 274–289. 10.1590/S0103-90162013000400009

[B24] Caulier S. Nannan C. Gillis A. Licciardi F. Bragard C. Mahillon J. (2019). Overview of the antimicrobial compounds produced by members of the *Bacillus subtilis* group. Front. Microbiol. 10:302. 10.3389/fmicb.2019.00302 30873135 PMC6401651

[B25] Chaparro J. M. Sheflin A. M. Manter D. K. Vivanco J. M. (2012). Manipulating the soil microbiome to increase soil health and plant fertility. Biol. Fertil. Soils 48, 489–499. 10.1007/s00374-012-0691-4

[B26] Chen Q. L. Cui H. L. Su J. Q. Penuelas J. Zhu Y. G. (2019). Antibiotic resistomes in plant microbiomes. Trends Plant Sci. 24, 530–541. 10.1016/j.tplants.2019.02.010 30890301

[B27] Compant S. Clément C. Sessitsch A. (2010). Plant growth-promoting bacteria in the rhizo- and endosphere of plants: their role in plant health. FEMS Microbiol. Ecol. 74, 1–13.20840217

[B28] de Souza R. S. C. Armanhi J. S. L. Arruda P. (2021). From microbiome to traits: designing synthetic microbial communities for improved crop resiliency. Front. Plant Sci. 12:735. 10.3389/fpls.2020.01179 32983187 PMC7484511

[B29] De-la-Vega-Camarillo E. Hernández-García J. A. Villa-Tanaca L. Hernández-Rodríguez C. (2023a). Unlocking the hidden potential of mexican teosinte seeds: revealing plant growth-promoting bacterial and fungal biocontrol agents. Front. Plant Sci. 14:1247814. 10.3389/fpls.2023.1247814 37860235 PMC10582567

[B30] De-la-Vega-Camarillo E. Sotelo-Aguilar J. Rios-Galicia B. Mercado-Flores Y. Arteaga-Garibay R. Villa-Tanaca L.. (2023b). Promotion of the growth and yield of *zea mays* by synthetic microbial communities from jala maize. Front. Microbiol. 14:1167839. 10.3389/fmicb.2023.1167839 37275168 PMC10235630

[B31] Delgado-Baquerizo M. Reich P. B. Trivedi C. Eldridge D. J. Abades S. Alfaro F. D.. (2020). Multiple elements of soil biodiversity drive ecosystem functions across biomes. Nat. Ecol. Evol. 4, 210–220. 10.1038/s41559-019-1084-y 32015427

[B32] Deng S. Wipf H. M. L. Pierroz G. Raab T. K. Khanna R. Coleman-Derr D. (2019a). A plant growth-promoting microbial soil amendment dynamically alters the strawberry root bacterial microbiome. Sci. Rep. 9:17677. 10.1038/s41598-019-53623-2 31776356 PMC6881409

[B33] Deng S. Wipf H. M. L. Pierroz G. Raab T. K. Khare E. Vogel J. P. (2019b). A plant growth-promoting microbial soil amendment dynamically alters the maize root bacterial microbiome. Sci. Rep. 9:14348. 10.1038/s41598-019-50832-9 31776356 PMC6881409

[B34] Deng Y. Jiang Y. H. Yang Y. He Z. Luo F. Zhou J. (2012). Molecular ecological network analyses. BMC Bioinformatics 13:113. 10.1186/1471-2105-13-113 22646978 PMC3428680

[B35] Ding G. C. Piceno Y. M. Heuer H. Weinert N. Dohrmann A. B. Carrillo A.. (2013). Changes of soil bacterial diversity as a consequence of agricultural land use in a semi-arid ecosystem. PLoS One 8:e59497. 10.1371/journal.pone.0059497 23527207 PMC3603937

[B36] Drusch M. Del Bello U. Carlier S. Colin O. Fernandez V. Gascon F.. (2012). Sentinel-2: ESA's optical high-resolution mission for gmes operational services. Remote Sens. Environ. 120, 25–36. 10.1016/j.rse.2011.11.026

[B37] Dubey A. Malla M. A. Khan F. Chowdhury K. Yadav S. Kumar A.. (2019). Soil microbiome: a key player for conservation of soil health under changing climate. Biodivers. Conserv. 28, 2405–2429. 10.1007/s10531-019-01760-5

[B38] Emam T. (2016). Local soil, but not commercial amf inoculum, increases native and non-native grass growth at a mine restoration site. Restor. Ecol. 24, 35–44. 10.1111/rec.12287

[B39] Faust K. Raes J. (2016). CoNet app: inference of biological association networks using cytoscape. F1000Res 5:1519. 10.12688/f1000research.9050.2 27853510 PMC5089131

[B40] Fierer N. Bradford M. A. Jackson R. B. (2007). Toward an ecological classification of soil bacteria. Ecology 88, 1354–1364. 10.1890/05-1839 17601128

[B41] Frac M. Hannula S. E. Bełka M. Jedryczka M. (2018). Fungal biodiversity and their role in soil health. Front. Microbiol. 9:707. 10.3389/fmicb.2018.00707 29755421 PMC5932366

[B42] Francioli D. Schulz E. Lentendu G. Wubet T. Buscot F. Reitz T. (2016). Mineral vs. organic amendments: microbial community structure, activity and abundance of agriculturally relevant microbes are driven by long-term fertilization strategies. Front. Microbiol. 7:1446. 10.3389/fmicb.2016.01446 27683576 PMC5022044

[B43] Friedman J. Alm E. J. (2012). Inferring correlation networks from genomic survey data. PLoS Comput. Biol. 8:e1002687. 10.1371/journal.pcbi.1002687 23028285 PMC3447976

[B44] Fukami J. Cerezini P. Hungria M. (2018). Azospirillum: benefits that go far beyond biological nitrogen fixation. AMB Express 8:73. 10.1186/s13568-018-0608-1 29728787 PMC5935603

[B45] Gao J. Luo Y. Wei Y. Huang Y. Zhang H. He W.. (2019). Effect of aridity and dune type on rhizosphere soil bacterial communities of *Caragana microphylla* in desert regions of Northern China. PLoS One 14:e0224195. 10.1371/journal.pone.0224195 31626675 PMC6799922

[B46] Gardes M. Bruns T. D. (1993). ITS primers with enhanced specificity for basidiomycetes - application to the identification of mycorrhizae and rusts. Mol. Ecol. 2, 113–118. 10.1111/j.1365-294X.1993.tb00005.x 8180733

[B47] Ge Y. Thomasson J. A. Sui R. (2011). Remote sensing of soil properties in precision agriculture: a review. Front. Earth Sci. 5, 229–238. 10.1007/s11707-011-0175-039808880

[B48] Geisseler D. Scow K. M. (2014). Long-term effects of mineral fertilizers on soil microorganisms —a review. Soil Biol. Biochem. 75, 54–63. 10.1016/j.soilbio.2014.03.023

[B49] Gorelick N. Hancher M. Dixon M. Ilyushchenko S. Thau D. Moore R. (2017). Google earth engine: planetary-scale geospatial analysis for everyone. Remote Sens. Environ. 202, 18–27. 10.1016/j.rse.2017.06.031

[B50] Gupta A. Singh U. B. Sahu P. K. Paul S. Kumar A. Malviya D.. (2022). Linking soil microbial diversity to modern agriculture practices: a review. Int. J. Environ. Res. Public Health 19:3141. 10.3390/ijerph19053141 35270832 PMC8910389

[B51] Hagberg A. Swart P. S. Chult D. (2008). Exploring Network Structure, Dynamics, and Function Using NetworkX. Los Alamos, NM: Los Alamos National Lab (LANL).

[B52] Harman G. E. Howell C. R. Viterbo A. Chet I. Lorito M. (2004). *Trichoderma* species—opportunistic, avirulent plant symbionts. Nat. Rev. Microbiol. 2, 43–56. 10.1038/nrmicro797 15035008

[B53] Hartmann M. Frey B. Mayer J. Mäder P. Widmer F. (2015). Distinct soil microbial diversity under long-term organic and conventional farming. ISME J. 9, 1177–1194. 10.1038/ismej.2014.210 25350160 PMC4409162

[B54] Hassan M. A. Yang M. Rasheed A. Yang G. Reynolds M. Xia X.. (2019). A rapid monitoring of NDVI across the wheat growth cycle for grain yield prediction using a multi-spectral UAV platform. Plant Sci. 282, 95–103. 10.1016/j.plantsci.2018.10.022 31003615

[B55] Hay R. K. M. (1995). Harvest index: a review of its use in plant breeding and crop physiology. Ann. Appl. Biol. 126, 197–216. 10.1111/j.1744-7348.1995.tb05015.x

[B56] Houbraken J. de Vries R. P. Samson R. A. (2014). Modern taxonomy of biotechnologically important *aspergillus* and *penicillium* species. Adv. Appl. Microbiol. 86, 199–249. 10.1016/B978-0-12-800262-9.00004-4 24377856

[B57] Hunter J. D. (2007). Matplotlib: a 2d graphics environment. Comput. Sci. Eng. 9, 90–95. 10.1109/MCSE.2007.55

[B58] International Organization for Standardization (2006). ISO 11464:2006 Soil Quality — Pretreatment of Samples for Physico-Chemical Analysis. Geneva: ISO.

[B59] IUSS Working Group WRB (2015). World Reference Base for Soil Resources 2014, Update 2015 International Soil Classification System for Naming Soils and Creating Legends for Soil Maps. World Soil Resources Reports No. 106. Rome: FAO.

[B60] Jaccard P. (1912). The distribution of the flora in the alpine zone. New Phytol. 11, 37–50. 10.1111/j.1469-8137.1912.tb05611.x

[B61] Jacoby R. Peukert M. Succurro A. Koprivova A. Kopriva S. (2017). The role of soil microorganisms in plant mineral nutrition—current knowledge and future directions. Front. Plant Sci. 8:1617. 10.3389/fpls.2017.01617 28974956 PMC5610682

[B62] Jones J. B. Wolf B. Mills H. A. (1991). Plant Analysis Handbook: A Practical Sampling, Preparation, Analysis, and Interpretation Guide. Athens, GA: Micro-Macro Publishing, Inc.

[B63] Jones J. B. Jr. (2001). Laboratory Guide for Conducting Soil Tests and Plant Analysis. Boca Raton, FL: CRC Preass. 10.1201/9781420025293

[B64] Kavamura V. N. Hayat R. Clark I. M. Rossmann M. Mendes R. Hirsch P. R.. (2018). Inorganic nitrogen application affects both taxonomical and predicted functional structure of wheat rhizosphere bacterial communities. Front. Microbiol. 9:1074. 10.3389/fmicb.2018.01074 29896167 PMC5986887

[B65] Keswani C. Prakash O. Bharti N. Vílchez J. I. Sansinenea E. Lally R. D.. (2019). Re-addressing the biosafety issues of plant growth promoting rhizobacteria. Sci. Total Environ. 690, 841–852. 10.1016/j.scitotenv.2019.07.046 31302549

[B66] Kielak A. M. Barreto C. C. Kowalchuk G. A. van Veen J. A. Kuramae E. E. (2016). The ecology of acidobacteria: moving beyond genes and genomes. Front. Microbiol. 7:744. 10.3389/fmicb.2016.00744 27303369 PMC4885859

[B67] Klindworth A. Pruesse E. Schweer T. Peplies J. Quast C. Horn M.. (2013). Evaluation of general 16s ribosomal rna gene pcr primers for classical and next-generation sequencing-based diversity studies. Nucleic Acids Res. 41:e1. 10.1093/nar/gks808 22933715 PMC3592464

[B68] Köberl M. Müller H. Ramadan E. M. Berg G. (2011). Desert farming benefits from microbial potential in arid soils and promotes diversity and plant health. PLoS ONE 6:e24452. 10.1371/journal.pone.0024452 21912695 PMC3166316

[B69] Kour D. Rana K. L. Yadav A. N. Yadav N. Kumar M. Kumar V.. (2020). Microbial biofertilizers: bioresources and eco-friendly technologies for agricultural and environmental sustainability. Biocatal. Agric. Biotechnol. 23:101487. 10.1016/j.bcab.2019.101487

[B70] Kurtz Z. D. Müller C. L. Miraldi E. R. Littman D. R. Blaser M. J. Bonneau R. A. (2015). Sparse and compositionally robust inference of microbial ecological networks. PLoS Comput. Biol. 11:e1004226. 10.1371/journal.pcbi.1004226 25950956 PMC4423992

[B71] Kuypers M. M. M. Marchant H. K. Kartal B. (2018). The microbial nitrogen-cycling network. Nat. Rev. Microbiol. 16, 263–276. 10.1038/nrmicro.2018.9 29398704

[B72] Larkin R. P. (2015). Soil health paradigms and implications for disease management. Annu. Rev. Phytopathol. 53, 199–221. 10.1146/annurev-phyto-080614-120357 26002292

[B73] Leff J. W. Jones S. E. Prober S. M. Barberán A. Borer E. T. Firn J. L.. (2015). Consistent responses of soil microbial communities to elevated nutrient inputs in grasslands across the globe. Proc. Natl. Acad. Sci. U.S.A. 112, 10967–10972. 10.1073/pnas.1508382112 26283343 PMC4568213

[B74] Legendre P. Gallagher E. D. (2001). Ecologically meaningful transformations for ordination of species data. Oecologia 129, 271–280. 10.1007/s004420100716 28547606

[B75] Lehmann A. Zheng W. Rillig M. C. (2017). Soil biota contributions to soil aggregation. Nat. Ecol. Evol. 1, 1828–1835. 10.1038/s41559-017-0344-y 29038473 PMC5701735

[B76] Lennon J. T. Jones S. E. (2011). Microbial seed banks: the ecological and evolutionary implications of dormancy. Nat. Rev. Microbiol. 9, 119–130. 10.1038/nrmicro2504 21233850

[B77] Li F. Chen L. Zhang J. Yin J. Huang S. (2020). *Mortierella elongata*'s roles in organic agriculture and crop growth promotion in a mineral soil. Land Degrad. Dev. 31, 881–891.

[B78] Li L. Hu Z. Tan G. Fan J. Chen Y. Xiao Y.. (2023). Enhancing plant growth in biofertilizer-amended soil through nitrogen-transforming microbial communities. Front. Plant Sci. 14:1259853. 10.3389/fpls.2023.1259853 38034579 PMC10683058

[B79] Lim Y. W. Kim B. K. Kim C. Jung H. S. Kim B.-S. Lee J.-H.. (2010). Assessment of soil fungal communities using pyrosequencing. J. Microbiol. 48, 284–289. 10.1007/s12275-010-9369-5 20571944

[B80] Lobell D. B. Bänziger M. Magorokosho C. Vivek B. (2011). Nonlinear heat effects on african maize as evidenced by historical yield trials. Nat. Clim. Chang. 1, 42–45. 10.1038/nclimate1043

[B81] Lori M. Hartmann M. Kundel D. Mayer J. Mueller R. C. Mäder P.. (2023). Soil microbial communities are sensitive to differences in fertilization intensity in organic and conventional farming systems. FEMS Microbiol. Ecol. 99:fiab033. 10.1093/femsec/fiad046 37160350 PMC10236208

[B82] Love M. I. Huber W. Anders S. (2014). Moderated estimation of fold change and dispersion for RNA-Seq data with DESeq2. Genome Biol. 15:550. 10.1186/s13059-014-0550-8 25516281 PMC4302049

[B83] Lozupone C. Lladser M. E. Knights D. Stombaugh J. Knight R. (2011). UniFrac: an effective distance metric for microbial community comparison. ISME J. 5, 169–172. 10.1038/ismej.2010.133 20827291 PMC3105689

[B84] Lupatini M. Korthals G. W. De Hollander M. Janssens T. K. S. Kuramae E. E. (2017). Soil microbiome is more heterogeneous in organic than in conventional farming system. Front. Microbiol. 7:2064. 10.3389/fmicb.2016.02064 28101080 PMC5209367

[B85] Ma A. Zhang J. Liu G. Zhuang X. Zhuang G. (2022). Cryosphere Microbiome Biobanks for Mountain Glaciers in China. Sustainability 14:2903. 10.3390/su14052903

[B86] Ma B.-L. Biswas D. K. (2015). “Precision nitrogen management for sustainable corn production,” in Sustainable Agriculture Reviews, eds. E. Lichtfouse and A. Goyal (Cham: Springer), 33–62.

[B87] Mahanty T. Bhattacharjee S. Goswami M. Bhattacharyya P. Das B. Ghosh A.. (2017). Biofertilizers: a potential approach for sustainable agriculture development. Environ. Sci. Pollut. Res. 24, 3315–3335. 10.1007/s11356-016-8104-0 27888482

[B88] Main-Knorn M. Pflug B. Louis J. Debaecker V. Müller-Wilm U. Gascon F. (2017). “Sen2Cor for Sentinel-2,” in Proceedings SPIE 10427, image and signal processing for remote sensing XXIII, 1042704.

[B89] Malusá E. Sas-Paszt L. Ciesielska J. (2012). Technologies for beneficial microorganisms inocula used as biofertilizers. Sci. World J. 2012:491206. 10.1100/2012/491206 22547984 PMC3324119

[B90] Mandic-Mulec I. Stefanic P. van Elsas J. D. (2015). Ecology of bacillaceae. Microbiol. Spectr. 3:TBS-0017-2013. 10.1128/microbiolspec.TBS-0017-2013 26104706

[B91] Maraveas C. (2022). Incorporating artificial intelligence technology in smart greenhouses: current state of the art. Appl. Sci. 13:14. 10.3390/app13010014

[B92] McKinney W. (2010). “Data structures for statistical computing in python,” in Proceedings of the 9th Python in Science Conference (Austin, TX), 51–56.

[B93] Mendes R. Kruijt M. de Bruijn I. Dekkers E. van der Voort M. Schneider J. H.. (2011). Deciphering the rhizosphere microbiome for disease-suppressive bacteria. Science 332, 1097–1100. 10.1126/science.1203980 21551032

[B94] Mitter E. K. Tosi M. Obregón D. Dunfield K. E. Germida J. J. (2021). Rethinking crop nutrition in times of modern microbiology: innovative biofertilizer technologies. Front. Sustain. Food Syst. 5:606815. 10.3389/fsufs.2021.606815

[B95] Mogili U. R. Deepak B. B. V. L. (2018). Review on application of drone systems in precision agriculture. Procedia Comput. Sci. 133, 502–509. 10.1016/j.procs.2018.07.063

[B96] Moll R. H. Kamprath E. J. Jackson W. A. (1982). Analysis and interpretation of factors which contribute to efficiency of nitrogen utilization. Agro. J. 74, 562–564. 10.2134/agronj1982.00021962007400030037x39414019

[B97] Moretti A. Logrieco A. F. Susca A. (2017). Mycotoxins: an underhand food problem. Methods Mol. Biol. 1542, 3–12. 10.1007/978-1-4939-6707-0_1 27924528

[B98] Nilsson R. H. Larsson K.-H. Taylor A. F. S. Bengtsson-Palme J. Jeppesen T. S. Schigel D.. (2019). The UNITE database for molecular identification of fungi: handling dark taxa and parallel taxonomic classifications. Nucleic Acids Res. 47, D259–D264. 10.1093/nar/gky1022 30371820 PMC6324048

[B99] Nosheen S. Ajmal I. Song Y. (2021). Microbes as biofertilizers, a potential approach for sustainable crop production. Sustainability 13:1868. 10.3390/su13041868

[B100] Olanrewaju O. S. Glick B. R. Babalola O. O. (2017). Mechanisms of action of plant growth-promoting bacteria. World J. Microbiol. Biotechnol. 33:197. 10.1007/s11274-017-2364-9 28986676 PMC5686270

[B101] Osono T. Takeda H. (2006). Fungal decomposition of abies needle and betula leaf litter. Mycologia 98, 172–179. 10.1080/15572536.2006.11832689 16894962

[B102] Panjaitan S. D. Dewi Y. S. K. Hendri M. I. Wicaksono R. A. Priyatman H. (2022). A drone technology implementation approach to conventional paddy fields application. IEEE Access 10, 120650–120658. 10.1109/ACCESS.2022.3221188

[B103] Parnell J. J. Berka R. Young H. A. Sturino J. M. Kang Y. Barnhart D. M.. (2016). From the lab to the farm: an industrial perspective of plant beneficial microorganisms. Front. Plant Sci. 7:1110. 10.3389/fpls.2016.01110 27540383 PMC4973397

[B104] Passioura J. B. (2006). Increasing crop productivity when water is scarce—from breeding to field management. Agric. Water Manag. 80, 176–196. 10.1016/j.agwat.2005.07.012

[B105] Paungfoo-Lonhienne C. Yeoh Y. K. Kasinadhuni N. R. P. Lonhienne T. G. A. Robinson N. Hugenholtz P.. (2015). Nitrogen fertilizer dose alters fungal communities in sugarcane soil and rhizosphere. Sci. Rep. 5:8678. 10.1038/srep08678 25728892 PMC5155403

[B106] Pedregosa F. Varoquaux G. Gramfort A. Michel V. Thirion B. Grisel O.. (2011). Scikit-learn: machine learning in python. *J. Mach. Learn*. Res. 12, 2825–2830.

[B107] Peigné J. Vian J.-F. Cannavacciuolo M. Lefevre V. Gautronneau Y. Boizard H. (2013). Assessment of soil structure in the transition layer between topsoil and subsoil using the profil cultural method. Soil Tillage Res. 127, 13–25. 10.1016/j.still.2012.05.014

[B108] Pérez-Pons M. E. Plaza-Hernández M. Alonso R. S. Parra-Domínguez J. Prieto J. (2020). Increasing profitability and monitoring environmental performance: a case study in the agri-food industry through an edge- IoT platform. Sustainability 13:283. 10.3390/su13010283

[B109] Piepho H. P. Büchse A. Richter C. (2004). A mixed modelling approach for randomized experiments with repeated measures. J. Agron. Crop Sci. 190, 230–247. 10.1111/j.1439-037X.2004.00097.x

[B110] Pisante M. Stagnari F. Grant C. A. (2012). Agricultural innovations for sustainable crop production intensification. Ital. J. Agron. 7:e40. 10.4081/ija.2012.e40

[B111] Pix4D SA (2020). Pix4Dmapper: Professional Drone Mapping and Photogrammetry Software. Lausanne: Pix4D SA.

[B112] QGIS Development Team (2024). QGIS Geographic Information System. Open Source Geospatial Foundation Project. Available online at: http://qgis.osgeo.org (accessed March 15, 2024).

[B113] Quast C. Pruesse E. Yilmaz P. Gerken J. Schweer T. Yarza P.. (2012). The SILVA ribosomal RNA gene database project: improved data processing and web-based tools. Nucleic Acids Res. 41, D590–D596. 10.1093/nar/gks1219 23193283 PMC3531112

[B114] R Core Team (2021). R: A Language and Environment for Statistical Computing. Vienna: R Foundation for Statistical Computing.

[B115] Raaijmakers J. M. Mazzola M. (2016). Soil immune responses. Science 352, 1392–1393. 10.1126/science.aaf3252 27313024

[B116] Radhakrishnan R. Hashem A. Abd_Allah E. F. (2017). *Bacillus*: a biological tool for crop improvement through bio-molecular changes in adverse environments. Front. Physiol. 8:667. 10.3389/fphys.2017.00667 28932199 PMC5592640

[B117] Reasoner D. J. Geldreich E. E. (1985). A new medium for the enumeration and subculture of bacteria from potable water. Appl. Environ. Microbiol. 49, 1–7. 10.1128/aem.49.1.1-7.1985 3883894 PMC238333

[B118] Rissanen A. J. Kurhela E. Aho T. Oittinen T. Tiirola M. (2010). Storage of environmental samples for guaranteeing nucleic acid yields for molecular microbiological studies. Appl. Microbiol. Biotechnol. 88, 977–984. 10.1007/s00253-010-2838-2 20730531

[B119] Rousk J. Bååth E. (2011). Growth of saprotrophic fungi and bacteria in soil. FEMS Microbiol. Ecol. 78, 17–30. 10.1111/j.1574-6941.2011.01106.x 21470255

[B120] Saleem M. Hu J. Jousset A. (2019). More than the sum of its parts: microbiome biodiversity as a driver of plant growth and soil health. Annu. Rev. Ecol. Evol. Syst. 50, 145–168. 10.1146/annurev-ecolsys-110617-062605

[B121] Sánchez B. Rasmussen A. Porter J. R. (2014). Temperatures and the growth and development of maize and rice: a review. Glob. Chang. Biol. 20, 408–417. 10.1111/gcb.12389 24038930

[B122] Schütz L. Gattinger A. Meier M. Müller A. Boller T. Mäder P.. (2018). Improving crop yield and nutrient use efficiency via biofertilization—a global meta-analysis. Front. Plant Sci. 8:2204. 10.3389/fpls.2017.02204 29375594 PMC5770357

[B123] scikit-bio Development Team (2020). scikit-bio: A Python package for bioinformatics and data science. Available online at: http://scikit-bio.org/

[B124] Seabold S. Perktold J. (2010). “Statsmodels: econometric and statistical modeling with python,” in Proceedings of the 9th Python in Science Conference (Austin, TX), 92–96.

[B125] Shade A. Peter H. Allison S. D. Baho D. L. Berga M. Bürgmann H.. (2012). Fundamentals of microbial community resistance and resilience. Front. Microbiol. 3:417. 10.3389/fmicb.2012.00417 23267351 PMC3525951

[B126] Singh R. P. Jha P. N. Jha P. N. (2020). The PGPR *Stenotrophomonas maltophilia* SBP-9 augments resistance against biotic and abiotic stress in wheat plants. Front. Microbiol. 8:1945. 10.3389/fmicb.2017.01945 29062306 PMC5640710

[B127] Song Q. Gao X. Song Y. Li Q. Chen Z. Li R.. (2023). Estimation and mapping of soil texture content based on unmanned aerial vehicle hyperspectral imaging. Sci Rep 13:4097. 10.1038/s41598-023-40384-2 37644047 PMC10465580

[B128] Späti K. Huber R. Finger R. (2021). Benefits of increasing information accuracy in variable rate technologies. Ecol. Econ. 185:107047. 10.1016/j.ecolecon.2021.107047

[B129] Spoorthi S. Shadaksharappa B. Suraj S. Manasa V. K. (2017). “Freyr drone: pesticide/fertilizers spraying drone - an agricultural approach,” in Proceedings of the 2017 2nd International Conference on Computing and Communications Technologies (ICCCT) (Chennai: IEEE) 252–255.

[B130] Sterkenburg E. Bahr A. Durling M. B. Clemmensen K. E. Lindahl B. D. (2015). Changes in fungal communities along a boreal forest soil fertility gradient. New Phytol. 207, 1145–1158. 10.1111/nph.13426 25952659

[B131] Suárez-Moreno Z. R. Caballero-Mellado J. Coutinho B. G. Mendonça-Previato L. James E. K. Venturi V. (2012). Common features of environmental and potentially beneficial plant-associated *Burkholderia*. Microb. Ecol. 63, 249–266. 10.1007/s00248-011-9929-1 21850446

[B132] Szilagyi-Zecchin V. J. Mógor Á. F. Figueiredo G. G. O. (2016). “Strategies for characterization of agriculturally important bacteria,” in Microbial Inoculants in Sustainable Agricultural Productivity, eds D. P. Singh, H. B. Singh, and R. Prabha (New Delhi: Springer India), 1–21.

[B133] Tedersoo L. Bahram M. Põlme S. Kõljalg U. Yorou N. S. Wijesundera R.. (2014). Global diversity and geography of soil fungi. Science 346:1256688. 10.1126/science.1256688 25430773

[B134] Tedersoo L. Smith M. E. (2013). Lineages of ectomycorrhizal fungi revisited: foraging strategies and novel lineages revealed by sequences from belowground. Fungal Biol. Rev. 27, 83–99. 10.1016/j.fbr.2013.09.001

[B135] Timmusk S. Behers L. Muthoni J. Muraya A. Aronsson A.-C. (2017). Perspectives and challenges of microbial application for crop improvement. Front. Plant Sci. 8:49. 10.3389/fpls.2017.00049 28232839 PMC5299024

[B136] Tripicchio P. Satler M. Dabisias G. Ruffaldi E. Avizzano C. A. (2015). “Towards smart farming and sustainable agriculture with drones,” in Proceedings of the 2015 International Conference on Intelligent Environments (Prague: IEEE), 140–143.

[B137] Trivedi P. Leach J. E. Tringe S. G. Sa T. Singh B. K. (2020). Plant–microbiome interactions: from community assembly to plant health. Nat. Rev. Microbiol. 18, 607–621. 10.1038/s41579-020-0412-1 32788714

[B138] Uddling J. Gelang-Alfredsson J. Piikki K. Pleijel H. (2007). Evaluating the relationship between leaf chlorophyll concentration and SPAD-502 chlorophyll meter readings. Photosynth Res. 91, 37–46. 10.1007/s11120-006-9077-5 17342446

[B139] van der Heijden M. G. A. Martin F. M. Selosse M. A. Sanders I. R. (2015). Mycorrhizal ecology and evolution: the past, the present, and the future. New Phytol. 205, 1406–1423. 10.1111/nph.13288 25639293

[B140] Van Elsas J. D. Chiurazzi M. Mallon C. A. Elhottovā D. Krištufek V. Salles J. F. (2012). Microbial diversity determines the invasion of soil by a bacterial pathogen. Proc. Natl. Acad. Sci. U.S.A. 109, 1159–1164. 10.1073/pnas.1109326109 22232669 PMC3268289

[B141] Van Rossum G. Drake F. L. (2009). Python 3 Reference Manual. Scotts Valley, CA: CreateSpace.

[B142] Vassilev N. Vassileva M. Nikolaeva I. (2006). Simultaneous P-solubilizing and biocontrol activity of microorganisms: potentials and future trends. Appl. Microbiol. Biotechnol. 71, 137–144. 10.1007/s00253-006-0380-z 16544140

[B143] Virtanen P. Gommers R. Oliphant T. E. Haberland M. Reddy T. Cournapeau D.. (2020). SciPy 1.0: fundamental algorithms for scientific computing in python. Nat. Methods 17, 261–272. 10.1038/s41592-020-0772-5 32015543 PMC7056644

[B144] Viscarra Rossel R. A. Behrens T. (2010). Using data mining to model and interpret soil diffuse reflectance spectra. Geoderma 158, 46–54. 10.1016/j.geoderma.2009.12.025

[B145] Viswanath G. K. Mukherjee A. Mukherjee A. (2021). “*Bacillus* as a potential phosphate solubilizing bacterium—an overview,” in Phosphorus in Soils: Biochemistry and Environmental Applications, eds. V. Kumar, A. K. Ghosh, and A. Kumar (Singapore: Springer), 363–377.

[B146] Vitousek P. M. Menge D. N. L. Reed S. C. Cleveland C. C. (2013). Biological nitrogen fixation: rates, patterns and ecological controls in terrestrial ecosystems. Philos. Trans. R. Soc. Lond. B Biol. Sci. 368:20130119. 10.1098/rstb.2013.0119 23713117 PMC3682739

[B147] Wagg C. Bender S. F. Widmer F. van der Heijden M. G. A. (2014). Soil biodiversity and soil community composition determine ecosystem multifunctionality. Proc. Natl. Acad. Sci. U.S.A. 111, 5266–5270. 10.1073/pnas.1320054111 24639507 PMC3986181

[B148] Wang C. Myint S. W. (2015). A simplified empirical line method of radiometric calibration for small unmanned aircraft systems-based remote sensing. IEEE J. Sel. Top. Appl. Earth Obs. Remote Sens. 8, 1876–1885. 10.1109/JSTARS.2015.2422716

[B149] Wang L. Tian Y. Yao X. Zhu Y. Cao W. (2014). Predicting grain yield and protein content in wheat by fusing multi-sensor and multi-temporal remote-sensing images. Field Crops Res. 164, 178–188. 10.1016/j.fcr.2014.05.00132704487

[B150] Waskom M. Botvinnik O. Gelbart M. Ostblom J. Hobson P. Lukauskas S.. (2020). mwaskom/seaborn: v0.11.0 (Sepetmber 2020). Zenodo. 10.5281/ZENODO.4019146

[B151] Waskom M. L. (2021). Seaborn: statistical data visualization. J. Open Source Softw. 6:3021. 10.21105/joss.03021

[B152] Watts S. C. Ritchie S. C. Inouye M. Holt K. E. (2019). FastSpar: rapid and scalable correlation estimation for compositional data. Bioinformatics 35, 1064–1066. 10.1093/bioinformatics/bty734 30169561 PMC6419895

[B153] Welles J. M. Norman J. M. (1991). Instrument for indirect measurement of canopy architecture. Agro. J. 83, 818–825. 10.2134/agronj1991.00021962008300050009x27120600

[B154] Wu L. Wen C. Qin Y. Yin H. Tu Q. Van Nostrand J. D.. (2015). Phasing amplicon sequencing on illumina miseq for robust environmental microbial community analysis. BMC Microbiol. 15:125. 10.1186/s12866-015-0450-4 26084274 PMC4472414

[B155] Wu S. C. Cao Z. H. Li Z. G. Cheung K. C. Wong M. H. (2005). Effects of biofertilizer containing N-fixer, P and K solubilizers and AM fungi on maize growth: a greenhouse trial. Geoderma 125, 155–166. 10.1016/j.geoderma.2004.07.003

[B156] Zhalnina K. Louie K. B. Hao Z. Mansoori N. da Rocha U. N. Shi S.. (2018). Dynamic root exudate chemistry and microbial substrate preferences drive patterns in rhizosphere microbial community assembly. Nat. Microbiol. 3, 470–480. 10.1038/s41564-018-0129-3 29556109

[B157] Zhang B. Penton C. R. Xue C. Wang Q. Zheng T. Tiedje J. M. (2015). Evaluation of the ion torrent personal genome machine for gene-targeted studies using amplicons of the nitrogenase gene nifH. Appl. Environ. Microbiol. 81, 4536–4545. 10.1128/AEM.00111-15 25911484 PMC4475867

[B158] Zhang C. Kovacs J. M. (2012). The application of small unmanned aerial systems for precision agriculture: a review. Precis. Agric. 13, 693–712. 10.1007/s11119-012-9274-531811713

[B159] Zheng B. X. Ding K. Yang X. R. Wadaan M. A. M. Hozzein W. N. Peñuelas J.. (2019). Straw biochar increases the abundance of inorganic phosphate solubilizing bacterial community for better rape (*brassica napus*) growth and phosphate uptake. Sci. Total Environ. 647, 1113–1120. 10.1016/j.scitotenv.2018.07.454 30180320

[B160] Zhu S. Vivanco J. M. Manter D. K. (2016). Nitrogen fertilizer rate affects root exudation, the rhizosphere microbiome and nitrogen-use-efficiency of maize. Appl. Soil Ecol. 107, 324–333. 10.1016/j.apsoil.2016.07.009

[B161] Zia S. Romano G. Spreer W. Sanchez C. Cairns J. Araus J. L.. (2013). Infrared thermal imaging as a rapid tool for identifying water-stress tolerant maize genotypes of different phenology. J. Agron. and Crop Sci. 199, 75–84. 10.1111/j.1439-037X.2012.00537.x

